# Synthesis of Pyrazole-Based Inhibitors of the Bacterial Enzyme *N*-Succinyl-l,l-2,6-Diaminopimelic Acid Desuccinylase (DapE) as Potential Antibiotics

**DOI:** 10.3390/ijms26010022

**Published:** 2024-12-24

**Authors:** Thomas DiPuma, Emma H. Kelley, Teerana Thabthimthong, Alayna Bland, Katherine Konczak, Katherine J. Torma, Thahani S. Habeeb Mohammad, Kenneth W. Olsen, Daniel P. Becker

**Affiliations:** Department of Chemistry and Biochemistry, Loyola University Chicago, 1032 West Sheridan Road, Chicago, IL 60660, USA; tdipuma@bu.edu (T.D.J.); ekelley2@luc.edu (E.H.K.); tthabthimthong@luc.edu (T.T.); abland@luc.edu (A.B.); kkonczak1@luc.edu (K.K.); ktorma@umich.edu (K.J.T.); thahanisifna@gmail.com (T.S.H.M.); kolsen@luc.edu (K.W.O.)

**Keywords:** DapE, metalloenzyme, antibiotic, ninhydrin

## Abstract

Based on the inhibitory potencies from earlier reported tetrazole thioether analogs, we now describe the synthesis and inhibition of pyrazole-based inhibitors of *N*-succinyl-l,l-2,6-diaminopimelic acid desuccinylase (DapE) from *Haemophilus influenzae* (*Hi*DapE). The most potent pyrazole analog **7d** bears an aminopyridine amide with an IC_50_ of 17.9 ± 8.0 μM, and the single enantiomer of ɑ-methyl analog **7q** has an IC_50_ of 18.8 µM, with potency residing in the (*R*)-enantiomer. Thermal shift revealed strong stabilization upon binding inhibitor **(R)-7q** with T_m_ = 50.2 °C and a K_i_ of 17.3 ± 2.8 μM. Enzyme kinetic experiments confirm competitive inhibition, and docking reveals key active site interactions.

## 1. Introduction

**Scheme 1:**
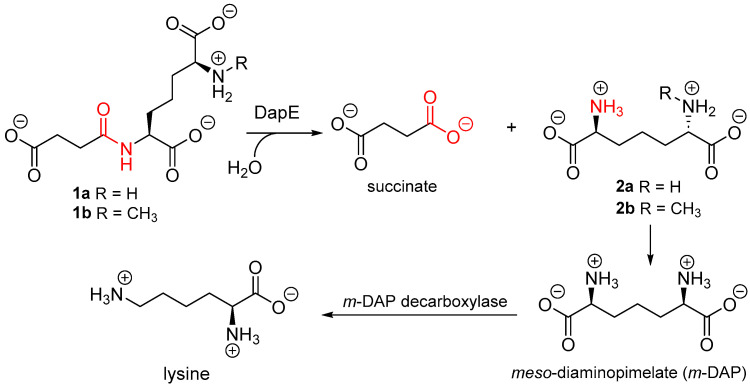
Enzymatic hydrolysis of endogenous substrate **1a** by DapE and conversion to lysine, and cleavage of synthetic DapE substrate analog **1b** used in the assay.

The persistent overuse of antibiotics has exacerbated the emergence of multidrug resistance within prokaryotic species, resulting in life-threatening ESKAPE pathogens, at the global expense of hospitalized patients [1,2]. In response, we have identified a previously underexplored bacterial enzyme target, *N*-Succinyl-l,l-diaminopimelic acid desuccinylase (DapE), toward the discovery of novel antibiotics. DapE plays an essential role in prokaryotic viability, as its purpose is to produce *m*-DAP and lysine, which are ultimately recruited for bacterial peptidoglycan cell wall synthesis [3], and lysine for peptide synthesis. DapE catalyzes the hydrolysis of *N*-succinyl-l,l-diaminopimelic acid (l,l-SDAP, **1a**) to succinic acid and l,l-diaminopimelate (DAP, **1b**) [4,5,6,7]. DAP acts as the precursor to form *m*-DAP, which is then converted to lysine (Scheme 1) [8,9,10]. Collectively, we have characterized the structural integrity of this enzyme and made significant strides towards elucidating the mechanism of action showcased through protein crystallography and molecular dynamics [11,12,13,14]. DapE is a promising new antibiotic target due to its absence in mammals, thus avoiding mechanism-based toxicity in humans [15]. Therefore, small-molecule inhibitors to DapE hold promise as novel antibiotics with a new mechanism of action.

**Figure 1:**
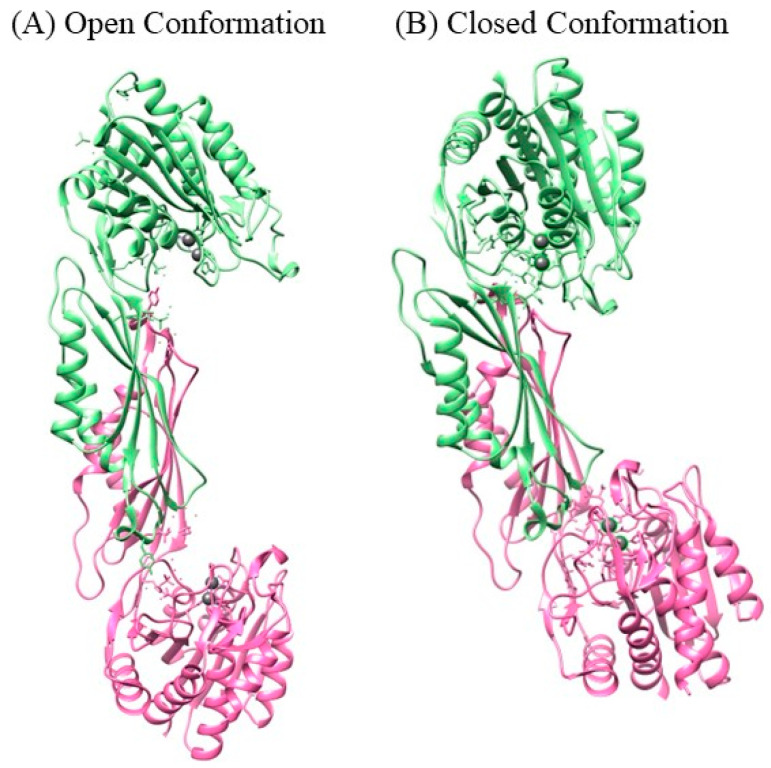
(**A**) Open conformation of *Nm*DapE enzyme (PDB 5UEJ) [12]. (**B**) Closed conformation of *Hi*DapE (PDB 5VO3) [11]. Subunit A is in light green and subunit B is in pink.

DapE is a hydrolase with a di-nuclear center consisting of two Zn(II) ions [11]. Consistent with the larger family of M20 metalloenzymes, DapE is homodimeric (41.6 kDa per subunit), with each subunit composed of catalytic and dimerization domain, both playing a critical role in catalysis. The catalytic domain houses the di-metallo active site consisting of the two Zn(II) ions. Elucidating the active site architecture through X-ray crystal structures of *Haemophilus influenza* (*Hi*DapE) showcased the substrate–enzyme interactions resulting in the observed dramatic conformational change upon substrate binding, as illustrated in Figure 1 [11]. Thus, binding of the substrate induces the dimer superstructure to flex and twist during catalysis, and this conformational change enables the enzyme to transform from the open conformation (PDB 5UEJ) to the closed conformation (PDB 5VO3), closing off access to the active site and driving the hydrolysis of the substrates scissile amide bond. In our previous published works, we have focused on the conformational mechanism and enzyme characterization of DapE [11] and synthesized inhibitors of DapE as potential antibiotics [13] utilizing a ninhydrin-based assay (**1b** to **2b** in Scheme 1) [14]. High-throughput screening previously revealed a tetrazole thioether as a DapE inhibitor, and a series of tetrazole analogs was synthesized to explore structure–activity relationships (SARs) as previously reported [13]. We selected pyrazoles because they are bioisosteres of tetrazoles and there are approved antibiotics such as cefoselis [16] and ceftolozane [17], along with other researchers synthesizing pyrazoles as potential antibiotics [18].

## 2. Results and Discussion

### 2.1. Synthesis and Inhibition of Pyrazole Analogs with HiDapE

**Scheme 2:**
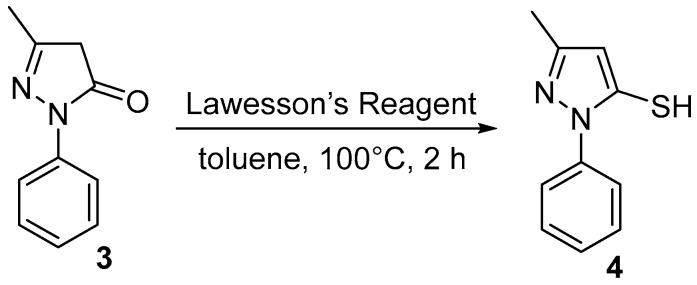
Synthesis of 3-methyl-1-phenyl-1*H*-pyrazole-5-thiol (**4**) [19].

**Scheme 3:**
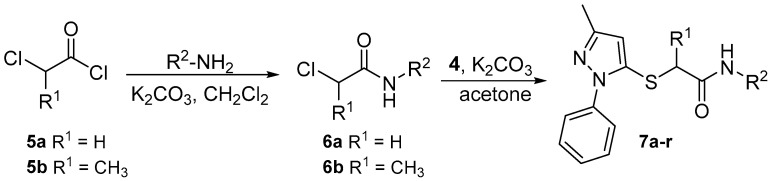
Synthesis of pyrazole derivatives **7a–s**.

Based on our earlier series of tetrazole thioether DapE inhibitors [13], we now report utilizing a bioisosteric pyrazole thioether scaffold to prepare an analogous series of DapE inhibitors. Preparation of 3-methyl-1-phenyl-1*H*-pyrazole-5-thiol (**4**) was performed by treating the commercially available edaravone (**3**) with Lawesson’s reagent [19] (Scheme 2). The α-chloro amides **6a** and **6b** were prepared from the requisite (hetero)aromatic amine and either chloroacetyl chloride (**5a**) or 2-chloropropionyl chloride (**5b**) in methylene chloride in the presence of potassium carbonate. These α-chloro intermediates were then reacted with 3-methyl-1-phenyl-1*H*-pyrazole-5-thiol (**4**) in acetone to afford the corresponding *N*-phenyl pyrazole thio-linked analogs (Scheme 3). All analogs synthesized were characterized with ^1^H and ^13^C NMR (Figures S1–S36). All tested derivatives that were solids exhibited melting points within a sharp 1–2 °C melting range (Table 1).

Inhibitory potencies of pyrazole analogs against HiDapE are shown in Table 1, including percent inhibition of DapE at 100 μM inhibitor, with IC_50_ values determined for several of the more potent inhibitors. (IC_50_ curves are available in the Supporting Information as Figures S37–S42.) Based on DapE inhibition at 100 μM, the most potent R^2^ substituents, first for R^1^ = H, are the six-membered (hetero)aryl groups including **7c** (R^2^ = phenyl, 80.5% inhibition), **7d** (R^2^ = 2-pyridyl, 85.7% inhibition), and **7h** (2-pyrazinyl, 96.2% inhibition). The same holds true for the derivatives bearing R^1^ = CH_3_, including **7n** (R^2^ = phenyl, 79.4% inhibition) and the very potent derivative **7o** (R^2^ = 2-pyridyl, 63.2% inhibition). The 2-thiazolyl analog **7a** is quite potent (IC_50_ = 22.4 µM), consistent with the earlier-reported tetrazoles, where R^2^ = 2-thiazolyl was the most potent derivative synthesized (72% inhibition at 100 μM; IC_50_ = 50.2 ± 5.0 μM) [13]. Yet, the 3-isoxazolyl analog **7b** was significantly less potent (20.3% inhibition at 100 µM).

Potencies for R^1^ = H were overall comparable to the pyrazole analogs oxazoles **7b** (R^1^ = H) and **7m** (R^1^ = racemic CH_3_) exhibit 20.3% and 19.5% inhibition at 100 μM, respectively. Additionally, *N*-phenyl analogs **7c** (R^1^ = H) and **7n** (R^1^ = racemic CH_3_) have 80.5% and 79.4% inhibition at 100 μM, respectively. To determine if DapE is selective for either epimer of R^1^ = CH_3_, we synthesized the enantiomers of the *N*-2-pyridyl pyrazole derivative **7p**, which as the racemate exhibited an IC_50_ of 35.6 μM. We found that (R)-pyrazole **7q** inhibits the enzyme almost completely at 100 μM (99% inhibition), whereas (S)-pyrazole (S)-**7r** inhibits the enzyme only by 14.6% at 100 μM, confirming that (R)-**7q** is the eutomer and (S)-**7r** is essentially inactive as the distomer. The IC_50_ value of the single enantiomer (R)-**7q** versus DapE is 18.8 μM (Figure S42), thus twice the potency of the racemic pyrazole **7o**, further confirming that this enantiomer is responsible for most if not all of the inhibitor potency of the racemat **7o**. The IC_50_ values were also determined for the most potent achiral R^1^ = H derivatives, with an IC_50_ of 17.9 +/− 8.0 μM for 2-pyridyl derivative **7d**, and an IC_50_ values of 20.2 ± 6.8 μM for 2-pyrazinyl derivatives **7h**.

### 2.2. Rate of DapE Hydrolysis in the Presence of Pyrazole **7q**

**Figure 2:**
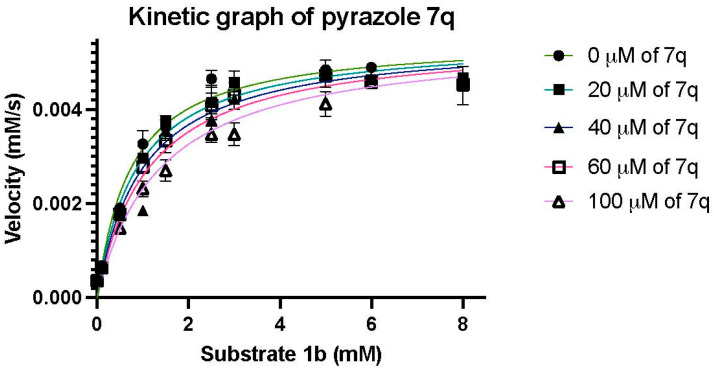
Kinetic graph of pyrazole (R)-**7q** with HiDapE.

We performed kinetic competition assays to investigate and confirm the binding mode of the pyrazole inhibitors. For these studies, we employed one of the most potent pyrazole analogs, single enantiomer **7q**. The velocity of the HiDapE enzyme was measured with different concentrations of substrate from 0 to 100 μM, and the plot is shown in Figure 2. After running the AICc statistical analysis [20,21] to compare noncompetitive and competitive models, it was determined that a competitive binding mode is over 100 times more likely than noncompetitive binding with a 99.2% probability.

A thermal shift assay [22] was conducted to observe changes in the thermal stability of DapE under various concentrations of the inhibitor (R)-**7q**, as summarized in Table 2. These studies were conducted on a Step One Real-Time PCR System^TM^ (Applied Biosystems, Waltham, MA, USA) and associated QuantStudio software (Version 7) through the fluorescence of the SYPRO Orange dye (Fisher Scientific, Waltham, MA, USA) during protein denaturation. We also used the thermal shift assay results to calculate the K_i_ value of pyrazole (R)-**7q**. We observed that at T_m_1 (50.2 °C), there was a stabilization of the enzyme when the inhibitor is binding, demonstrated by the +13 °C positive shift in the melt temperature as the inhibitor increases in concentration. This indicates that the inhibitor is binding primarily to the native unfolded state of the enzyme [23,24]. Additionally, we observed a subsequent T_m_2 at 78.0 °C involving a destabilization of the enzyme, as shown by the approximate −3 °C negative shift in melt temperature as the inhibitor increases in concentration, indicating at this elevated temperature that the inhibitor is binding to primarily the molten globule state [23,24]. We have previously observed the second melt temperature with the DapE enzyme [25]. From the positive melt temperature shift data at T_m_1, we were able to calculate a K_i_ value for (R)-**7q** of 17.3 +/− 2.8 μM (Figure S44). Achiral pyrazole **7d** has a comparable IC_50_ value of IC_50_ = 17.9 ± 8.0 μM.

### 2.3. Molecular Docking

**Figure 3:**
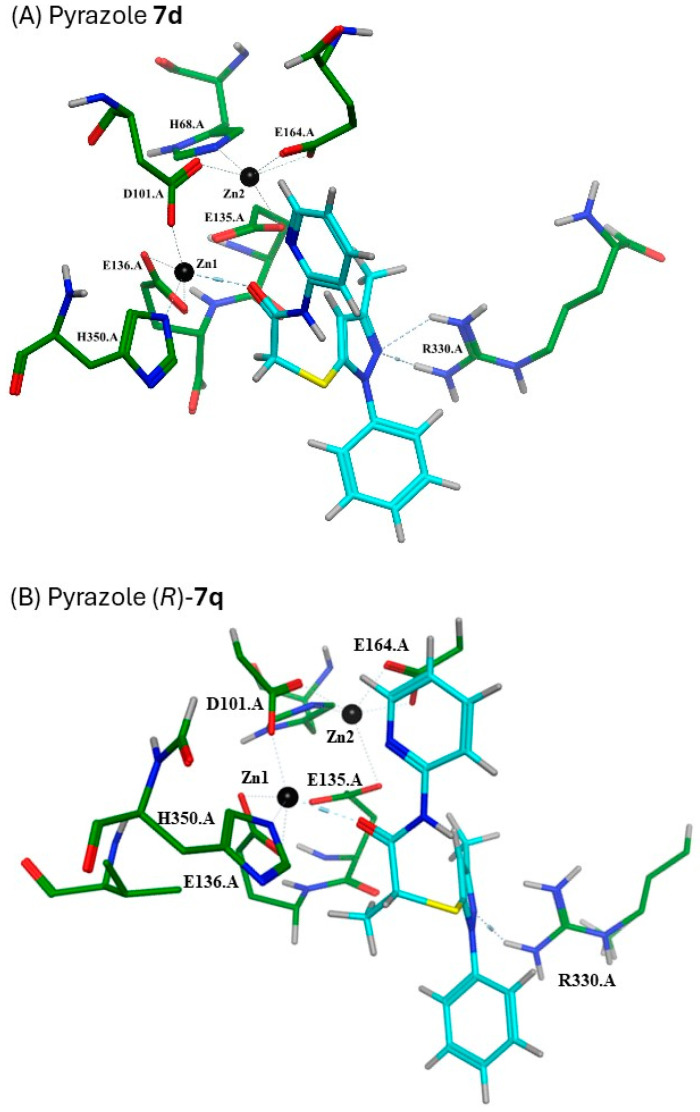

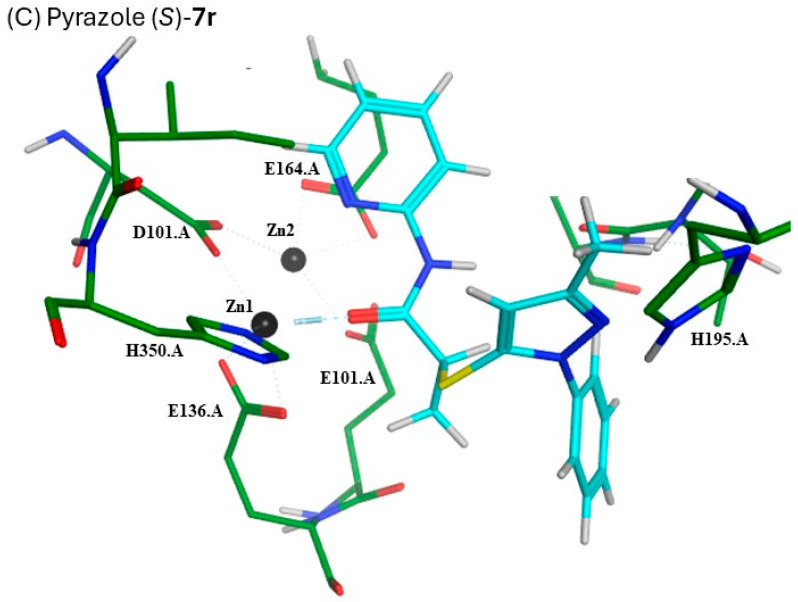
Docking of (**A**) most achiral pyrazole **7d**, (**B**) (R)-pyrazole **7q**, and (**C**) (S)-pyrazole **7r** to the active site of NmDapE, PDB 5UEJ.

To gain further insight into the possible binding modes of the pyrazole analogs to DapE, we performed docking experiments utilizing the open form of DapE from Neisseria meningitidis (NmDapE, PDB 5UEJ) with the most potent pyrazole analog, the achiral (R^1^ = H) 2-pyridyl derivative **7d**, as well as with the single enantiomers, eutomer (R)-**7q** (S-score = −9.4) and distomer **(S)-7r** (R^1^ = CH_3_, S-score = −8.2). More stable, low-energy poses showcased key interactions, as illustrated in Figure 3. For the most potent pyrazole **7d**, the amide carbonyl oxygen interacts with one of the zinc atoms, while N2 of the pyrazole ring participates in hydrogen bonding with Arg330A. Compared to docking of the tetrazole reported earlier [13] there appears to be a new interaction at the N2 position on the pyrazole ring compared to the interactions observed with the respective tetrazole compound. We observed similar interactions with the potent enantiomer (R)-**7q**; specifically, we observed the same key hydrogen bond of Arg330A hydrogen bonding with the N2 of the pyrazole ring. We did not observe the same binding interaction with (S)-**7r**.

## 3. Materials and Methods

### 3.1. Synthesis

All solvents were distilled prior to use or purchased as anhydrous grade. All reagents were used without further purification unless otherwise noted. Molecular sieves were activated at 300–350 °C under vacuum unless otherwise stated. Chloroacetyl chloride, 2-chloropropionyl chloride, amino heterocycles, amines, and potassium carbonate were purchased from Sigma-Aldrich (St. Louis, MO, USA). Synthesis of 3-methyl-1-phenyl-1*H*-pyrazole-5-thiol was conducted according to the literature. All synthetic reactions were conducted under an atmosphere of nitrogen. Silica gel 60 Å, 40–75 µm (200 × 400 mesh) was used for column chromatography. Aluminum-backed silica gel 200 µm plates purchased from Sorbtech (Norcross, GA, USA) were used for TLC. ^1^H NMR and ^13^C NMR spectra were obtained using a Bruker 500 MHz spectrometer (Bruker Corporation, Madison, WI, USA) with tetramethylsilane (TMS) as the internal standard. The ^1^H NMR spectra were collected using the 500 MHz frequency and the ^13^C NMR spectra were obtained using the 125 MHz frequency of the same Bruker 500 MHz spectrometer. NMR spectra were processed using Mnova NMR software (version 4.14.1) produced by Mestrelab Research (Santiago de Compostela, Spain). The purity of all compounds was determined to be ≥95% by high-performance liquid chromatography (HPLC). HRMS spectra were measured on a TOF instrument by electrospray ionization (ESI). HRMS spectra were collected using a Waters Acquity I class UPLC and Xevo G2-XS QT of mass spectrometer (Waters Xevo, Wexford, Ireland) with Waters Acquity BEH C18 column (1.7 µm, 2.1 × 50 mm; Waters Xevo, Wexford, Ireland).

General procedure for 2-chloro-*N*-acetamides and 2-((1-phenyl-1*H*-tetrazol-5-yl)thio)-*N*-acetamides in thioether derivative synthesis. To a tall clear 4-dram vial, equipped with a magnetic stir rod/flea bar, the desired amine (1.0 eq) along with potassium carbonate (1.2 eq) was suspended in anhydrous dichloromethane (0.3 M) freshly distilled over calcium hydride. The reaction vessel was purged with nitrogen gas to ensure the transformation took place under inert atmospheric conditions. To the reaction vessel, chloroacetyl chloride or 2-chloropropionyl chloride (3.5 eq) was added dropwise under nitrogen at 0 °C to room temperature, as the contents were set to stir at 250 rpm paired with frequent degassing of the vessel being essential with periodic HPLC monitoring. Upon completion of reaction, dichloromethane and excess acid chloride was concentrated under reduced pressure, and the resultant crude was suspended in DI water (2 mL). The organic product was extracted using ethyl acetate (3 × 2 mL), washed with brine (3 × 2mL), and the combined organic layers were dried over anhydrous Na_2_SO_4_. Then, the solvent was removed by evaporation under reduced pressure yielding a crude product mixture, which was subject to purification through recrystallization with a two-solvent system of a 1:3 ratio of dichloromethane and hexane to afford the corresponding 2-chloro-*N*-acetamide, which was isolated and reacted for the next step.

In a clear 2-dram vial equipped with a magnetic stir rod/flea bar, the isolated amide intermediate (1.0 eq), phenyl tetrazole thiol (1.0 eq), and potassium carbonate (1.2 eq) was suspended in anhydrous acetone (0.3 M) under nitrogen at reflux for 30min to an hour with periodic HPLC monitoring. Upon completion, acetone was evaporated off under reduced pressure and the resultant crude was suspended in DI water (2 mL). The organic product was extracted using ethyl acetate (3 × 2 mL), washed with brine (3 × 2 mL), and the combined organic layers were dried over anhydrous Na_2_SO_4_. The solvent was then removed by evaporation under reduced pressure yielding a crude product mixture, which was subjected to purification through recrystallization with a di-solvent system of a 1:3 ratio dichloromethane and hexane to afford the corresponding 2-((1-phenyl-1*H*-tetrazol-5-yl)thio)-*N*-acetamide.

2-((3-Methyl-1-phenyl-1*H*-pyrazol-5-yl)thio)-*N*-(thiazol-2-yl)acetamide (**7a**). 2-Chloro-*N*-2-thiazolylacetamide [26] intermediate (30.0 mg, 0.163 mmol) was used in this reaction. The crude mixture of **7a** was purified with DCM/hexane (1:3) recrystallization solvents to afford **7a** as a white solid (30.7 mg, 57%); mp: 157–159 °C. ^1^H NMR (500 MHz, CDCl_3_) δ 11.2 (s, 1H), 7.53–7.46 (m, 2H), 7.44–7.36 (m, 2H), 7.39–7.29 (m, 2H), 7.00 (d, *J* = 3.6 Hz, 1H), 6.34 (s, 1H), 3.55 (s, 2H), 2.28 (s, 3H). ^13^C NMR (126 MHz, CDCl_3_) δ 165.5, 158.6, 149.9, 139.0, 136.8, 132.3, 128.9, 128.1, 125.3, 125.1, 114.1, 112.0, 77.3, 77.0, 76.8, 39.1, 13.6. HRMS (ESI): C_15_H_15_N_4_OS_2_ [M+H]^+^: calcd: 331.0687; found: 331.0682.

*N*-(Isoxazol-3-yl)-2-((3-methyl-1-phenyl-1*H*-pyrazol-5-yl)thio)acetamide (**7b**). 2-Chloro-*N*-3-isoxazolylacetamide intermediate [27] (30.0 mg, 0.186 mmol) was used in this reaction. The crude mixture of **7b** was purified with DCM/hexane (1:3) recrystallization solvents to afford **7b** as a white solid (44.8 mg, 77%); mp: 119–121 °C. ^1^H NMR (500 MHz, CDCl_3_) δ 9.15 (s, 1H), 8.29 (dd, *J* = 1.8, 0.6 Hz, 1H), 7.54–7.48 (m, 2H), 7.48–7.41 (m, 2H), 7.41–7.34 (m, 1H), 6.99 (d, *J* = 1.8 Hz, 1H), 6.32 (s, 1H), 3.50 (s, 2H), 2.29 (s, 3H). ^13^C NMR (126 MHz, CDCl_3_) δ 165.6, 159.1, 156.8, 150.0, 139.0, 132.3, 129.1, 128.3, 125.3, 111.34, 99.1, 77.3, 77.0, 76.8, 39.6, 13.6. HRMS (ESI): C_15_H_15_N_4_O_2_S [M+H]^+^: calcd: 315.0916; found: 315.0909.

2-((3-Methyl-1-phenyl-1*H*-pyrazol-5-yl)thio)-*N*-phenylacetamide (**7c**). Chloroacetanilide intermediate [28] (30.0 mg, 0.176 mmol) was used in this reaction. The crude mixture of **7c** was purified by column chromatography using ethyl acetate/hexane (30/70) to afford **7c** as a white solid (66%); mp: oil. ^1^H NMR (500 MHz, CDCl_3_) δ 8.16 (s, 1H), 7.55–7.47 (m, 2H), 7.44 (dd, J = 8.6, 6.8 Hz, 2H), 7.40–7.24 (m, 5H), 7.12 (tt, J = 7.2, 1.4 Hz, 1H), 6.32 (s, 1H), 3.49 (s, 2H), 2.29 (s, 3H), 1.76–1.66 (m, 0H), 1.33 (s, 2H), 0.93–0.79 (m, 2H). ^13^C NMR (126 MHz, CDCl_3_) δ 165.1, 150.2, 139.3, 139.0, 137.1, 133.1, 130.9, 129.1, 128.9, 128.8, 128.1, 125.2, 124.8, 119.8, 114.1, 111.0, 77.3, 77.0, 76.7, 66.2, 40.1, 38.8, 37.1, 36.6, 33.8, 33.2, 31.9, 30.1, 30.0, 29.6, 29.6, 29.5, 29.3, 29.1, 28.9, 27.0, 26.7, 25.9, 24.6, 23.3, 22.6, 14.1, 13.6, 10.8. HRMS (ESI): C_18_H_18_N_3_OS [M+H]^+^: calcd: 324.1171; found: 324.1165.

2-((3-Methyl-1-phenyl-1*H*-pyrazol-5-yl)thio)-*N*-(pyridine-2-yl)acetamide (**7d**). 2-(Chloroacetylamino)pyridine intermediate [29,30] (30.0 mg, 0.175 mmol) was used in the reaction. The crude mixture of **7d** was purified by column chromatography using ethyl acetate/hexane (30/70) to afford **7d** as a brown oil (17.5 mg, 31%); mp: oil. ^1^H NMR (500 MHz, CDCl_3_) δ 8.73 (s, 1H), 8.28 (ddd, *J* = 4.9, 2.0, 0.9 Hz, 1H), 8.11 (d, *J* = 8.4 Hz, 1H), 7.70 (ddd, *J* = 8.5, 7.3, 1.9 Hz, 1H), 7.55–7.49 (m, 2H), 7.43 (dd, *J* = 8.5, 7.0 Hz, 2H), 7.39–7.32 (m, 1H), 7.07 (ddd, *J* = 7.4, 4.9, 1.1 Hz, 1H), 6.33 (s, 1H), 3.49 (s, 2H), 2.28 (s, 3H), 1.26 (s, 1H). ^13^C NMR (126 MHz, CDCl_3_) δ 165.8, 150.6, 149.9, 147.9, 139.0, 138.3, 132.7, 129.0, 128.0, 125.2, 120.2, 113.8, 111.1, 77.2, 77.0, 76.7, 40.0, 29.7, 13.6. HRMS (ESI): C_17_H_17_N_4_OS [M+H]+: calcd: 325.1123; found: 325.1124.

2-((3-Methyl-1-phenyl-1*H*-pyrazol-5-yl)thio)-*N*-(5-methylpyridin-2-yl)acetamide (**7e**). 2-Chloro-*N*-(5-methyl-2-pyridinyl)acetamide intermediate [30] (30.0 mg, 0.161 mmol) was used in this reaction. The crude mixture of **7e** was purified by column chromatography using ethyl acetate/hexane (30/70) to afford **7e** as a white solid (22.4 mg, 41%); mp: 114–115 °C. ^1^H NMR (500 MHz, CDCl_3_) δ 8.71 (s, 1H), 8.09 (d, *J* = 2.3 Hz, 1H), 8.01 (d, *J* = 8.4 Hz, 1H), 7.56–7.48 (m, 3H), 7.43 (dd, *J* = 8.7, 7.0 Hz, 2H), 7.39–7.32 (m, 1H), 6.32 (s, 1H), 3.49 (s, 2H), 2.29 (d, *J* = 12.5 Hz, 6H), 1.26 (d, *J* = 3.9 Hz, 1H). ^13^C NMR (126 MHz, CDCl_3_) δ 165.6, 149.9, 148.5, 147.8, 139.1, 138.9, 132.9, 129.7, 129.0, 128.0, 125.2, 113.3, 111.0, 77.2, 77.0, 76.7, 39.9, 29.7, 17.8, 13.6, 1.0. HRMS (ESI): C_18_H_19_N_4_OS [M+H]^+^: calcd: 339.1280; found: 339.1271.

*N*-(5-Chloropyridin-2-yl)-2-((3-methyl-1-phenyl-1*H*-pyrazol-5-yl)thio)acetamide (**7f**). 2-Chloro-*N*-(5-chloro-2-pyridinyl)acetamide [31] (30.0 mg, 0.146 mmol) was used in this reaction. The crude mixture of **7f** was purified with DCM/hexane (1:3) recrystallization solvents to afford **7f** as a white solid (17.4 mg, 33%); mp: 72–73 °C. ^1^H NMR (500 MHz, CDCl_3_) δ 8.70 (s, 1H), 8.22 (d, J = 2.5 Hz, 1H), 8.09 (d, J = 8.9 Hz, 1H), 7.66 (dd, J = 8.8, 2.6 Hz, 1H), 7.57–7.48 (m, 2H), 7.43 (dd, J = 8.7, 6.9 Hz, 2H), 7.39–7.32 (m, 1H), 6.32 (s, 1H), 3.48 (s, 2H), 2.29 (s, 3H). ^13^C NMR (126 MHz, CDCl_3_) δ 165.8, 150.0, 148.9, 146.7, 146.5, 139.0, 138.1, 137.9, 132.5, 129.0, 128.1, 127.3, 125.2, 114.5, 114.5, 111.3, 77.2, 77.0, 76.7, 42.7, 40.0, 13.6. HRMS (ESI): C_17_H_16_ClN_4_OS [M+H]^+^: calcd: 359.0733; found: 359.0759.

2-((3-Methyl-1-phenyl-1*H*-pyrazol-5-yl)thio)-*N*-(pyrimidin-2-yl)acetamide (**7g**). 2-Chloro-*N*-2-pyrimidinylacetamide intermediate [32] (30.0 mg, 0.194 mmol) was used in this reaction. The crude mixture of **7g** was purified with DCM/hexane (1:3) recrystallization solvents to afford **7g** as a white solid (46.2 mg, 81%); mp: 159–161 °C. ^1^H NMR (500 MHz, CDCl_3_δ 9.34 (s, 1H), 8.54 (d, *J* = 4.9 Hz, 2H), 7.49–7.43 (m, 2H), 7.37–7.30 (m, 2H), 7.30–7.23 (m, 1H), 6.95 (t, *J* = 4.9 Hz, 1H), 6.31 (s, 1H), 3.88 (s, 2H), 2.21 (s, 3H). ^13^C NMR (126 MHz, CDCl_3_) δ 158.3, 156.9, 149.7, 139.3, 128.8, 127.8, 125.2, 116.5, 111.6, 77.2, 77.0, 76.7, 40.6, 13.6. HRMS (ESI): C_16_H_16_N_5_OS [M+H]^+^: calcd: 326.1076; found: 326.1072.

2-((3-Methyl-1-phenyl-1*H*-pyrazol-5-yl)thio)-*N*-(pyrazin-2-yl)acetamide (**7h**). 2-Chloro-*N*-2-pyrazinylacetamide intermediate [33] (30.0 mg, 0.174 mmol) was used in this reaction. The crude mixture of **7h** was purified by column chromatography using ethyl acetate/hexane (30/70) to afford **7h** as a brown oil (19.6 mg, 35%); mp: oil. ^1^H NMR (500 MHz, CDCl_3_) δ 9.42 (s, 1H), 8.63 (s, 1H), 8.37 (d, J = 2.6 Hz, 1H), 8.24 (dd, J = 2.6, 1.6 Hz, 1H), 7.55–7.48 (m, 2H), 7.42 (dd, J = 8.7, 7.0 Hz, 2H), 7.38–7.30 (m, 1H), 6.35 (s, 1H), 3.51 (s, 2H), 2.29 (s, 3H). ^13^C NMR (126 MHz, CDCl_3_) δ 165.8, 150.0, 147.3, 142.0, 140.7, 138.9, 136.7, 132.2, 129.0, 128.1, 125.2, 111.5, 77.2, 77.0, 76.7, 65.8, 39.9, 31.9, 30.1, 29.7, 29.3, 22.7, 15.2, 14.1, 13.6. HRMS (ESI): C_16_H_16_N_5_OS [M+H]^+^: calcd: 326.1076; found: 326.1078.

*N*-(6-Chloropyrazin-2-yl)-2-((3-methyl-1-phenyl-1*H*-pyrazol-5-yl)thio)acetamide (**7i**). 2-Chloro-*N*-(6-chloro-2-pyrazinyl)acetamide [34] (30.0 mg, 0.146 mmol) intermediate was used in this reaction. The crude mixture of **7i** was purified by column chromatography using ethyl acetate/hexane (30/70) to afford **7i** as an oil (46%); mp: oil. ^1^H NMR (500 MHz, CDCl_3_) δ 9.31 (s, 2H), 8.57 (s, 2H), 8.37 (d, J = 0.6 Hz, 2H), 7.52–7.46 (m, 4H), 7.45–7.38 (m, 4H), 7.38–7.31 (m, 2H), 6.35 (s, 2H), 3.50 (s, 4H), 2.30 (s, 6H), 1.28 (dd, J = 11.3, 2.2 Hz, 1H), 1.28 (s, 1H), 1.26 (s, 1H), 0.91–0.82 (m, 1H). ^13^C NMR (126 MHz, CDCl_3_) δ 165.9, 150.1, 146.3, 146.2, 139.8, 138.8, 133.8, 131.9, 129.1, 128.1, 125.3, 111.8, 77.2, 77.0, 76.7, 40.1, 31.5, 29.7, 22.6, 14.1, 13.6. HRMS (ESI): C_16_H_15_ClN_5_OS [M+H]^+^: calcd: 360.0686; found: 360.0689.

*N*,*N*-Diethyl-2-((3-methyl-1-phenyl-1*H*-pyrazol-5-yl)thio)acetamide (**7j**). The chloro-*N*,*N*-diethylacetamide intermediate [35] (30.0mg, 0.147 mmol) was used in this reaction. The crude mixture of **7j** was purified after workup to afford **7j** as a clear oil (18.2 mg, 30%); mp: oil. ^1^H NMR (500 MHz, CDCl_3_) δ 7.61–7.55 (m, 2H), 7.48–7.41 (m, 2H), 7.45–7.32 (m, 1H), 7.26 (s, 1H), 6.34 (d, J = 1.5 Hz, 1H), 3.50 (d, J = 1.6 Hz, 2H), 3.32 (qd, J = 7.2, 1.6 Hz, 2H), 3.15 (qd, J = 7.2, 1.5 Hz, 2H), 2.32 (d, J = 1.6 Hz, 3H), 1.25 (s, 0H), 1.08 (td, J = 7.1, 1.3 Hz, 6H). ^13^C NMR (126 MHz, CDCl_3_) δ 166.3, 149.6, 139.4, 133.9, 128.8, 128.7, 127.7, 125.6, 125.1, 111.9, 77.2, 77.0, 76.7, 42.4, 40.5, 38.2, 29.7, 14.2, 13.6, 12.8. HRMS (ESI): C_16_H_22_N_3_OS [M+H]^+^: calcd: 304.1484; found: 303.1477.

*N*-(1H-Benzo[d]imidazol-2-yl)-2-((3-methyl-1-phenyl-1*H*-pyrazol-5-yl)thio)acetamide (**7k**). 2-(Chloroacetamido)benzimidazole intermediate [36] (30.0 mg, 0.143 mmol) was used in this reaction. The crude mixture of **7k** was purified with DCM/hexane (1:3) recrystallization solvents to afford **7k** as a white solid (26.8 mg, 52%); mp: 174–176 °C. ^1^H NMR (500 MHz, CDCl_3_) δ 11.83 (s, 2H), 7.46 (dd, *J* = 7.7, 1.6 Hz, 2H), 7.40 (dt, *J* = 7.5, 3.7 Hz, 2H), 7.33–7.19 (m, 5H), 6.27 (s, 1H), 3.62 (s, 2H), 2.24 (s, 3H). ^13^C NMR (126 MHz, CDCl_3_) δ 168.8, 149.7, 147.5, 139.1, 132.3, 128.7, 127.7, 125.1, 122.8, 112.5, 77.2, 77.0, 76.7, 39.8, 29.7, 13.6. HRMS (ESI): C_19_H_18_N_5_OS [M+H]^+^: calcd: 364.1232; found: 364.1223.

2-((3-Methyl-1-phenyl-1*H*-pyrazol-5-yl)thio)-*N*-(thiazol-2-yl)propanamide (**7l**). 2-Chloro-*N*-(thiazol-2-yl)propanamide intermédiate [37] (30.0 mg, 0.157mmol) was used in this reaction. The crude mixture of **7l** was purified with DCM/hexane (1:3) recrystallization solvents to afford **7l** as an oil (31.3 mg, 60%); mp: oil. ^1^H NMR (500 MHz, CDCl_3_) δ 10.99 (s, 1H), 7.47–7.41 (m, 2H), 7.39–7.23 (m, 4H), 6.99 (d, *J* = 3.6 Hz, 1H), 6.34 (s, 1H), 3.61 (q, *J* = 7.1 Hz, 1H), 2.27 (s, 3H), 1.45 (d, *J* = 7.1 Hz, 3H). ^13^C NMR (126 MHz, CDCl_3_) δ 168.8, 158.5, 149.7, 139.0, 136.9, 130.1, 128.7, 127.8, 125.5, 114.3, 113.9, 77.2, 77.0, 76.7, 47.1, 31.5, 22.6, 17.2, 14.1, 13.6. HRMS (ESI): C_16_H_17_N_4_OS_2_ [M+H]^+^: calcd: 345.0844; found: 345.0856.

*N*-(Isoxazol-3-yl)-2-((3-methyl-1-phenyl-1*H*-pyrazol-5-yl)thio)propanamide (**7m**). 2-chloro-*N*-(isoxazol-3-yl)propanamide intermediate [38] (30.0 mg, 0.206mmol) was used in this reaction. The crude mixture of **7m** was purified with DCM/hexane (1:3) recrystallization solvents to afford **7m** as a white solid (27.0 mg, 40%); mp: 98–100 °C. ^1^H NMR (500 MHz, CDCl_3_) δ 9.10 (s, 1H), 8.27 (d, *J* = 1.7 Hz, 1H), 7.51–7.44 (m, 2H), 7.43–7.36 (m, 2H), 7.36–7.30 (m, 1H), 6.97 (d, *J* = 1.8 Hz, 1H), 6.35 (s, 1H), 3.57 (q, *J* = 7.2 Hz, 1H), 2.29 (s, 3H), 1.43 (d, *J* = 7.2 Hz, 3H). ^13^C NMR (126 MHz, CDCl_3_) δ 169.2, 159.2, 158.8, 157.0, 149.7, 139.0, 130.7, 128.8, 128.0, 125.5, 113.6, 99.2, 99.0, 77.2, 77.0, 76.7, 48.1, 22.1, 17.2, 13.6. HRMS (ESI): C_16_H_17_N_4_O_2_S[M+H]^+^: calcd: 329.1072; found: 329.1055.

2-((3-Methyl-1-phenyl-1*H*-pyrazol-5-yl)thio)-*N*-phenylpropanamide (**7n**). 2-Chloro-*N*-phenylpropanamide intermediate [39] (40.0 mg, 0.217 mmol) was used in this reaction. The crude mixture of **7n** was purified with DCM/hexane (1:3) recrystallization solvents to afford **7n** as a white solid (26.0 mg, 31%); mp: 132–134 °C. ^1^H NMR (500 MHz, CDCl_3_δ 7.82 (s, 1H), 7.52–7.46 (m, 2H), 7.43 (ddd, *J* = 7.9, 6.9, 1.4 Hz, 2H), 7.43–7.33 (m, 1H), 7.36–7.26 (m, 4H), 7.11 (tt, *J* = 6.9, 1.6 Hz, 1H), 6.36 (s, 1H), 3.58 (q, *J* = 7.3 Hz, 1H), 2.29 (s, 3H), 2.29 (s, 1H), 1.47 (d, *J* = 7.2 Hz, 3H). ^13^C NMR (126 MHz, CDCl_3_) δ 168.7, 149.9, 139.1, 137.2, 131.7, 128.9, 128.0, 125.4, 124.6, 119.7, 112.7, 77.2, 77.0, 76.7, 49.1, 17.7, 13.6. HRMS (ESI): C_19_H_20_N_3_OS [M+H]^+^: calcd: 338.1327; found: 338.1306.

*N*-Benzyl-2-((3-methyl-1-phenyl-1*H*-pyrazol-5-yl)thio)propanamide (**7o**). 2-Chloro-*N*-(phenylmethyl)propanamide intermediate [40] (30.0 mg, 0.151 mmol) was used in this reaction.The crude mixture of **7o** was purified with DCM/hexane (1:3) recrystallization solvents to afford **7o** as a white solid (23.9 mg, 37%); mp: 97–99 °C. ^1^H NMR (500 MHz, CDCl_3_) δ 7.47–7.37 (m, 4H), 7.40–7.32 (m, 1H), 7.31 (dddd, *J* = 11.0, 6.9, 4.3, 2.3 Hz, 3H), 7.14–7.08 (m, 2H), 6.38 (t, *J* = 5.6 Hz, 1H), 6.21 (s, 1H), 4.33 (dd, *J* = 14.8, 6.0 Hz, 1H), 4.27 (dd, *J* = 14.7, 5.7 Hz, 1H), 3.54 (q, *J* = 7.3 Hz, 1H), 2.29 (s, 3H), 1.43 (d, *J* = 7.3 Hz, 3H), 1.32–1.22 (m, 0H). ^13^C NMR (126 MHz, CDCl_3_) δ 170.6, 149.8, 139.1, 137.7, 132.4, 128.9, 128.7, 127.9, 127.6, 127.6, 125.2, 112.0, 77.2, 77.0, 76.7, 48.1, 43.7, 22.3, 17.9, 13.6. HRMS (ESI): C_20_H_22_N_3_OS [M+H]^+^: calcd: 352.1484; found: 352.1485.

2-((3-Methyl-1-phenyl-1*H*-pyrazol-5-yl)thio)-*N*-(pyridine-2-yl)propanamide (**7p**). 2-chloro-*N*-2-pyridinyl-propanamide intermediate [41] (30.0 mg, 0.192 mmol) was used in this reaction. The crude mixture of **7p** was purified with DCM/hexane (1:3) recrystallization solvents to afford **7p** as a white solid (38.6 mg, 45%); mp: 130–132 °C. ^1^H NMR (500 MHz, CDCl_3_) δ 8.42 (s, 1H), 8.26 (ddd, *J* = 4.9, 2.0, 0.9 Hz, 1H), 8.10–8.04 (m, 1H), 7.68 (ddd, *J* = 8.7, 7.2, 2.0 Hz, 1H), 7.51–7.45 (m, 2H), 7.42–7.35 (m, 2H), 7.34–7.27 (m, 1H), 7.05 (ddd, *J* = 7.3, 4.9, 1.0 Hz, 1H), 6.36 (s, 1H), 3.54 (q, *J* = 7.2 Hz, 1H), 2.28 (s, 3H), 1.44 (d, *J* = 7.2 Hz, 3H). ^13^C NMR (126 MHz, CDCl_3_) δ 169.4, 150.8, 149.7, 147.8, 139.1, 138.2, 131.2, 128.8, 127.9, 125.5, 120.0, 113.8, 113.2, 77.2, 77.0, 76.7, 48.7, 17.4, 13.6. HRMS (ESI): C_18_H_19_N_4_OS [M+H]^+^: calcd: 339.1280; found: 339.1289.

(*R*)-2-((3-Methyl-1-phenyl-1*H*-pyrazol-5-yl)thio)-*N*-(pyridine-2-yl)propanamide (**7q**). (*S*)-2-chloro-*N*-(pyridin-2-yl)propenamide intermediate [41] (16.0 mg, 0.09 mmol) was used in this reaction. The crude mixture of **7q** was purified with DCM/hexane (1:3) recrystallization solvents to afford **7q** as a white solid (30.0 mg, 85%); mp: 130–132 °C. ^1^H NMR (500 MHz, CDCl_3_) δ 8.31 (s, 1H), 8.00 (dd, *J* = 8.4, 1.1 Hz, 1H), 7.62 (ddd, *J* = 8.7, 7.3, 1.9 Hz, 1H), 7.45–7.37 (m, 2H), 7.35–7.29 (m, 2H), 7.29–7.20 (m, 1H), 6.98 (ddd, *J* = 7.4, 4.9, 1.0 Hz, 1H), 6.29 (s, 1H), 3.47 (q, *J* = 7.2 Hz, 1H), 2.21 (s, 3H), 1.37 (d, *J* = 7.2 Hz, 3H). ^13^C NMR (126 MHz, CDCl_3_) δ 169.4, 150.7, 149.8, 147.9, 139.1, 138.2, 131.2, 128.8, 127.9, 125.5, 120.1, 113.8, 113.2, 48.7, 17.5, 13.6. HRMS (ESI): C_18_H_19_N_4_OS [M+H]^+^: calcd: 339.1280; found: 339.1287.

(*S*)-2-((3-Methyl-1-phenyl-1*H*-pyrazol-5-yl)thio)-*N*-(pyridine-2-yl)propanamide (**7r**). (*R*)-2-Chloro-*N*-(pyridin-2-yl)propenamide intermediate [41] (50.0 mg, 0.27 mmol) was used in this reaction. The crude mixture of **7r** was purified with DCM/hexane (1:3) recrystallization solvents to afford **7r** as a white solid (91.4 mg, 20%); mp: 130–132 °C. ^1^H NMR (500 MHz, CDCl_3_) δ 8.32 (s, 1H), 8.01 (dd, *J* = 8.4, 1.1 Hz, 1H), 7.62 (ddd, *J* = 8.7, 7.3, 1.9 Hz, 1H), 7.45–7.38 (m, 2H), 7.36–7.29 (m, 2H), 7.29–7.21 (m, 1H), 6.99 (ddd, *J* = 7.4, 4.9, 1.0 Hz, 1H), 6.30 (s, 1H), 3.48 (q, *J* = 7.2 Hz, 1H), 2.22 (s, 3H), 1.38 (d, *J* = 7.2 Hz, 3H). ^13^C NMR (126 MHz, CDCl_3_) δ 169.4, 150.7, 149.7, 147.9, 139.1, 138.2, 131.2, 128.8, 127.9, 125.5, 120.1, 113.8, 113.2, 48.7, 17.5, 13.6. HRMS (ESI): C_18_H_19_N_4_OS [M+H]^+^: calcd: 339.1280; found: 339.1278.

### 3.2. Enzymatic Assays

Inhibitory Assay Protocol: A discontinuous inhibition assay was performed utilizing a Techne PCR Thermal Cycler System [14,25]. The volume of each component was adjusted to fit a total reaction volume of 100 μL. The final potential inhibitor concentration was 100 μM for pyrazoles **7a**–**7r** for screening experiments or 5–200 μM for IC_50_ experiments. All potential inhibitors were dissolved in neat dimethylsulfoxide (DMSO), and the pre-assay concentrations were adjusted to give a final concentration of 5% DMSO in the assay. The final *Hi*DapE concentration was 8 nM. To a 50 mM HEPES (pH 7.5) buffered solution at 30 °C was added the selected inhibitor followed by *Hi*DapE and incubated for 10 min. *N*^6^-Methyl-**l**,**l**-SDAP (2 mM) was added, and the mixture was allowed to react for 10 min, followed by heating at 100 °C for 1 min and then cooled to 0 °C for 1 min. A 2% ninhydrin solution (100 μL) was added, and the mixture was mixed well by rapid pipetting while cooled at 0 °C. The reaction was then heated at 80 °C for 15 min. The absorbance of an 80 μL aliquot was recorded at 570 nm on a BioTek Synergy 2 microplate reader (Biotek Instruments, Winooski, VT, USA). All inhibition assays were performed in triplicate. The data were then exported and analyzed using Prism (version 10.4.1, GraphPad Prism, Boston, MA, USA).

Kinetic assay Protocol: A discontinuous kinetic assay was performed utilizing a Techne PCR Thermal Cycler System [14,25]. The volume of each component was adjusted to fit a total reaction volume of 100 μL. The final potential inhibitor concentration was 0, 20, 40, 60, and 100 μM for pyrazole **7r**, where the inhibitor was dissolved in dimethylsulfoxide (DMSO), and the pre-assay concentrations were adjusted to give a final concentration of 5% DMSO in the assay. The in-assay *Hi*DapE concentration was 8 nM. To a 50 mM HEPES (pH 7.5) buffered solution at 30 °C was added the selected inhibitor followed by HiDapE and incubated for 10 min. *N*^6^-Methyl-l,l-SDAP at varied concentrations (0–8 mM) were added, and the mixture was allowed to react for 10 min, followed by heating at 100 °C for 1 min and then cooled to 0 °C for 1 min. A 2% ninhydrin solution (50 μL) was added, and the mixture was mixed well by rapid pipetting while cooled at 0 °C. The reaction was then heated at 80 °C for 15 min. The absorbance of an 80 μL aliquot was recorded at 570 nm on a BioTek Synergy 2 microplate reader. All concentrations were performed in triplicate. The data were then exported and analyzed using Prism using a non-linear regression Michaelis–Menten equation and using AICc statistical analysis to determine which inhibitory binding mode was preferred.

Thermal Shift Assay Protocol: Thermal shift studies were conducted on a Step One Real-Time PCR System and the associated QuantStudio software. Protein denaturation was measured by detecting the change in fluorescence of the SYPRO Orange dye. *Hi*DapE was used at a final concentration of 200 nM, and SYPRO Orange was used at a final concentration of 10×. The experiment was carried out in 10 μL triplicates in 50 mM HEPES buffer at pH 7.5 in nanopure water, and the inhibitor concentrations were selected based on the half-maximal inhibitory concentration (IC_50_) values of the inhibitor. Sample solutions were dispensed into a 96-well optical reaction plate (Thermo Fisher Scientific, Kalamazoo, MI, USA), and the plate was sealed with an optical PCR plate sheet (Thermo Fisher Scientific, Kalamazoo, MI, USA). The assay was run according to Bhayani [22]. Melting curves were obtained from the negative derivative and exported from the instrument to Microsoft Excel (Version 2402). The negative first derivatives of the melting curves were differentiated into the third derivative. T_m_ values were plotted against the log [concentration], and the K_i_ values were calculated using a derived Van’t Hoff equation according to Bhayani [22]

Docking Protocol: Molecular models of pyrazole analogs **7d**, **7q**, and **7r** were built using the Molecular Operating Environment (MOE, Version 2024.06) computational suite [42]. The ligands were protonated at 310 K, pH 7.4 and a salt value of 0.1, followed by energy minimization in the gas phase using the MMFF94x force field [43]. A database file of each ligand was generated following initial preparation. Further optimization of the ligands were performed using the MOE’s database wash utility with enumerate protonation at pH 7.4 with a limit of 10. The X-ray crystal structure of *Nm*DapE (PDB 5UEJ), which was already optimized and protonated at 310 K, pH 7.4, and a salt value of 0.1 using Protonate3D, was then uploaded into MOE and prepared for docking using MOE’s Structure Preparation utility. The active site of chains A and B, which includes the dinuclear Zn(II) center, was surveyed using MOE’s Site Finder utility and populated with inactivated dummy atoms to define the docking location. Following preparation of the ligands and the *Nm*DapE docking receptor model, induced-fit molecular docking using the previously generated ligand conformation database was carried out with the solvent atoms inactivated at the docking site specified by the dummy atoms populating the substrate-binding cleft of chain A and B of the docking receptor. The alpha triangle placement method with affinity dG scoring generated 1000 poses, which were further refined using the induced fit method with GBVI/WSA dG scoring to obtain the top 100 poses. The Amber14:EHT [44] force field was used to perform these calculations. We were able to run docking experiments with *Nm*DapE rather than *Hi*DapE, which was used for the assay, because the sequence homology of *Nm*DapE and *Hi*Dape is >99.99%.

## 4. Conclusions

DapE represents a promising but underexplored bacterial enzyme target toward the discovery of antibiotics with a new mechanism of action, which is critical in addressing the ever-increasing global cases of antibiotic-resistant bacterial infections. We have explored the synthesis and SAR of a series of pyrazoles derived as bioisosteres of the earlier described tetrazole DapE inhibitors. The most potent tetrazole reported earlier has an IC_50_ of 50.2 µM, whereas the present pyrazoles include several analogs with potencies of 20 µM, including achiral pyridyl analog **7d** with IC_50_ = 17.9 µM and chiral pyridyl analog **7q** with IC_50_ = 18.8 µM. Furthermore, kinetic enzyme competition experiments confirm that these pyrazole inhibitors are competitive inhibitors. Thermal shift assays confirm pyrazole inhibitors stabilize the enzyme, which is consistent with previous results [25], and we calculated a K_i_ = 17.3 for chiral pyridyl analog **7q** and K_i_ = 17.1 achiral pyridyl analog **7d**. Docking also reveals key interactions with the active site, specifically the hydrogen bonding interaction between the *N*2 of the pyrazole and R330.A. This highly druggable molecular template will serve as a productive lead scaffold for the next generation of DapE inhibitors.

## Figures and Tables

**Table 1:** Physiochemical data and inhibitory potencies of pyrazole thioether analogs **7a–r**.

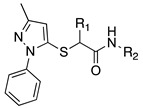						
**Compound**	**R^1^**	**R^2^**	**M.W.** **(g/mol)**	**cLogP**	**Melting** **Point (°C)**	**IC_50_ (μM) or % Inhibition at 100 μM**
**7a**	-H	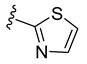	330.4	2.7	157–159	95.2 ± 0.6% IC_50_ = 22.4 ± 4.9 μM
**7b**	-H	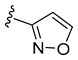	314.4	2.3	119–121	20.3 ± 3.6%
**7c**	-H	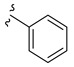	323.4	3.3	Oil	80.5 ± 3.0%
**7d**	-H	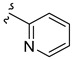	324.4	2.7	Oil	85.7 ± 1.2% IC_50_ = 17.9 ± 8.0 μM
**7e**	-H	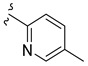	338.4	3.0	114–115	64.3 ± 5.5%
**7f**	-H	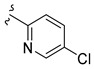	358.8	3.2	72–73	54.2 ± 0.3%
**7g**	-H	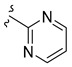	325.4	2.2	159–161	32.4 ± 3.4%
**7h**	-H	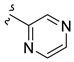	325.4	1.9	Oil	96.2% ± 4.0 IC_50_ = 20.2 ± 6.8 μM
**7i**	-H	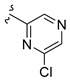	359.8	2.5	Oil	99.7 ± 0.4% IC_50_ = 43.6 ± 9.1μM
**7j**	-H	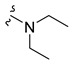	303.4	3.0	Oil	47.4 ± 7.2%
**7k**	-H	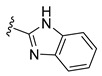	363.4	3.0	174–176	36.9 ± 2.7%
**7l**	-CH_3_	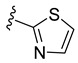	344.5	2.3	Oil	28.0 ± 1.8%
**7m**	-CH_3_	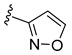	328.4	2.8	98–100	19.5 ± 0.1%
**7n**	-CH_3_	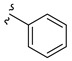	337.4	3.8	132–134	79.4 ± 3.6%
**7o**	-CH_3_	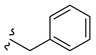	351.5	3.9	97–99	63.2 ± 1.8%
**7p**	-CH_3_	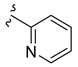	338.4	3.3	130–132	97.4 ± 2.0% IC_50_ = 35.7 ± 6.8 μM
**7q**	-(*R*)-CH_3_	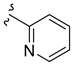	338.4	3.3	130–131	99.1 ± 1.6% IC_50_ = 18.8 ± 4.3 μM
**7r**	-(*S*)-CH_3_	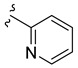	338.4	3.3	130–132	14.6 ± 9.5%

**Table 2:** Thermal shift data with HiDapE against pyrazole (R)-**7q** with 200 nM enzyme, 10X dye.

Thermal Shift Experiment	T_m_1 (*Hi*DapE) [°C]
No inhibitor	50.2 +/− 0.2
1 μM	50.8 +/− 0.6
5 μM	50.1 +/− 0.1
10 μM	50.2 +/− 0.1
20 μM	52.7 +/− 0.3
40 μM	55.7 +/− 0.3
60 μM	60.6 +/− 0.7
80 μM	61.3 +/− 0.5
100 μM	63.1 +/− 0.2

## Data Availability

Data are contained within this article and Supplementary Material. Samples of the compounds described may be available from the authors.

## Supplementary Materials

The following supporting information can be downloaded at: https://www.mdpi.com/article/10.3390/ijms26010022/s1.
