# Supplementary material for: Synthesis of Pyrazole-Based Inhibitors of the Bacterial Enzyme N-Succinyl-l,l-2,6-Diaminopimelic Acid Desuccinylase (DapE) as Potential Antibiotics
Source: Int J Mol Sci. 2024 Dec 24;26(1):22. doi: 10.3390/ijms26010022 (PMC11720106; doi:10.3390/ijms26010022)
Supplement: Supplementary file 1 [file ijms-26-00022-s001.zip › ijms-3351085-supplementary.pdf]

Supplemental Information

Design And Synthesis of Pyrazole-based Inhibitors of the Bacterial Enzyme *N*-Succinyl-L,L-2,6-Diaminopimelic Acid Desuccinylase as Potential Antibiotics

Thomas DiPuma[#,a], Emma H. Kelley[#, a], Teerana Thabthimthong[a], Alayna Bland[a], Katherine Konczak[a], Claire Herbert[a], Thahani Sifna[a], Kenneth W. Olsen[a], and Daniel P. Becker[a*]

[a]Department of Chemistry and Biochemistry, Loyola University Chicago, 1032 West Sheridan Road, Chicago, IL 60660, USA

Table of Contents

Spectral Data

- **Compound 7a:**
  - S1: ^{1}H NMR Spectrum ....Page 3
  - S2: ^{13}C NMR Spectrum .... Page 4
- **Compound 7b:**
  - S3: ^{1}H NMR Spectrum .... Page 5
  - S4: ^{13}C NMR Spectrum .... Page 6
- **Compound 7c:**
  - S5: ^{1}H NMR Spectrum .... Page 7
  - S6: ^{13}C NMR Spectrum .... Page 8
- **Compound 7d:**
  - S7: ^{1}H NMR Spectrum .... Page 9
  - S8: ^{13}C NMR Spectrum .... Page 10
- **Compound 7e:**
  - S9: ^{1}H NMR Spectrum .... Page 11
  - S10: ^{13}C NMR Spectrum .... Page 12
- **Compound 7f:**
  - S11: ^{1}H NMR Spectrum .... Page 13
  - S12: ^{13}C NMR Spectrum .... Page 14
- **Compound 7g:**
  - S13: ^{1}H NMR Spectrum .... Page 15
  - S14: ^{13}C NMR Spectrum .... Page 16
- **Compound 7h:**
  - S15: ^{1}H NMR Spectrum .... Page 17
  - S16: ^{13}C NMR Spectrum .... Page 18
- **Compound 7i:**
  - S17: ^{1}H NMR Spectrum .... Page 19
  - S18: ^{13}C NMR Spectrum .... Page 20

- **Compound 7j:**
    - S19: ^{1}H NMR Spectrum .... Page 21
    - S20: ^{13}C NMR Spectrum .... Page 22
- **Compound 7k:**
    - S21: ^{1}H NMR Spectrum .... Page 23
    - S22: ^{13}C NMR Spectrum .... Page 24
- **Compound 7l:**
    - S23: ^{1}H NMR Spectrum .... Page 25
    - S24: ^{13}C NMR Spectrum .... Page 26
- **Compound 7m:**
    - S25: ^{1}H NMR Spectrum .... Page 27
    - S26: ^{13}C NMR Spectrum .... Page 28
- **Compound 7n:**
    - S27: ^{1}H NMR Spectrum .... Page 29
    - S28: ^{13}C NMR Spectrum .... Page 30
- **Compound 7o:**
    - S29: ^{1}H NMR Spectrum .... Page 31
    - S30: ^{13}C NMR Spectrum .... Page 32
- **Compound 7p:**
    - S31: ^{1}H NMR Spectrum .... Page 33
    - S32: ^{13}C NMR Spectrum .... Page 34
- **Compound 7q:**
    - S33: ^{1}H NMR Spectrum .... Page 35
    - S34: ^{13}C NMR Spectrum .... Page 36
- **Compound 7r:**
    - S35: ^{1}H NMR Spectrum .... Page 37
    - S36: ^{13}C NMR Spectrum .... Page 38

Enzymatic Assays

- Inhibitory Graphs:
    - S37: Pyrazole **7a** .... Page 39
    - S38: Pyrazole **7d** .... Page 39
    - S39: Pyrazole **7f** .... Page 40
    - S40: Pyrazole **7h** .... Page 40
    - S41: Pyrazole **7p** .... Page 41
    - S42: Pyrazole **7r** .... Page 41
- Thermal Shift Assay:
    - S43: Pyrazole **7d** .... Page 42
    - S44: Pyrazole **7r** .... Page 42

2-((3-methyl-1-phenyl-1*H*-pyrazol-5-yl)thio)-*N*-(thiazol-2-yl)acetamide (7a)

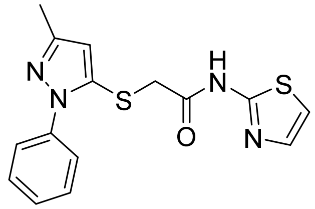

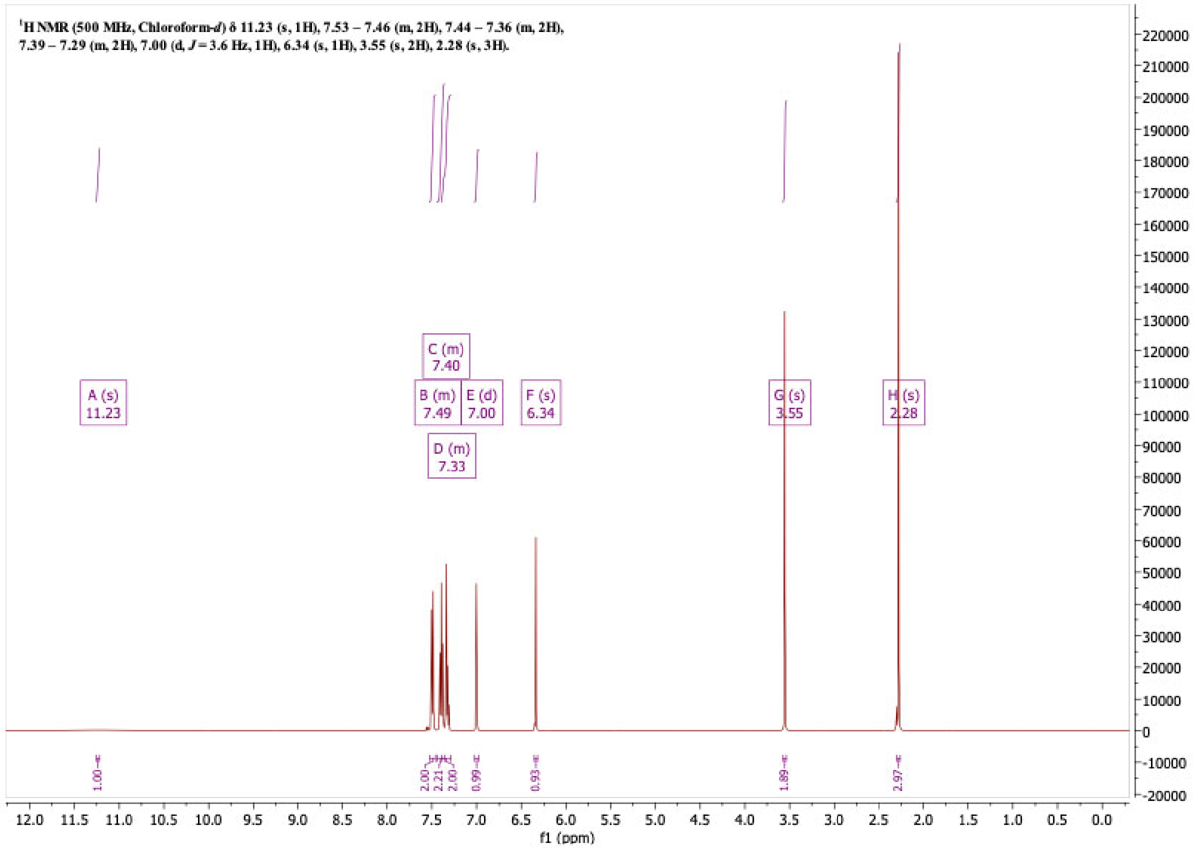

S1: ^{1}H NMR spectrum of 2-((3-methyl-1-phenyl-1*H*-pyrazol-5-yl)thio)-*N*-(thiazol-2-yl)acetamide (**7a**).

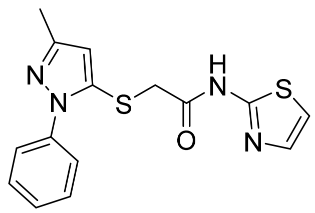

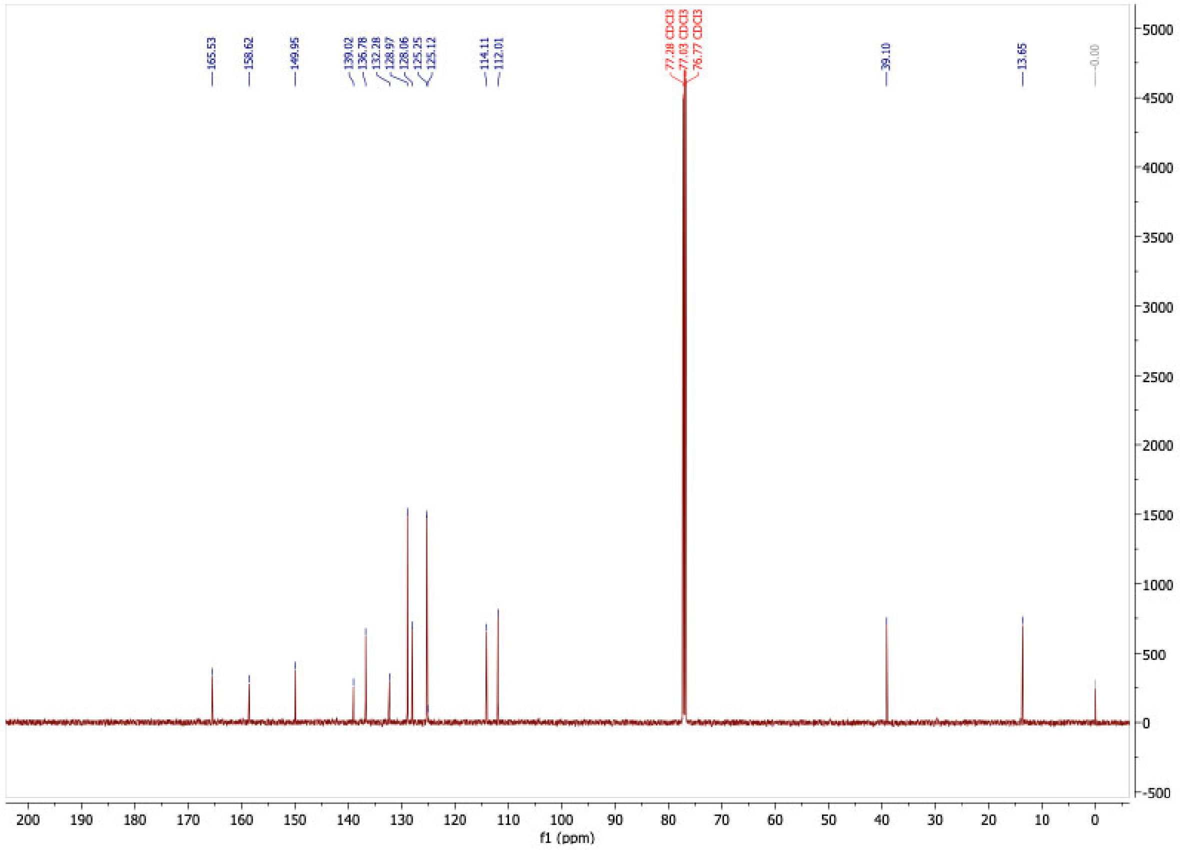

S2: ^{13}C NMR spectrum of -((3-methyl-1-phenyl-1*H*-pyrazol-5-yl)thio)-*N*-(thiazol-2-yl)acetamide (**7a**).

_N_-(isoxazol-3-yl)-2-((3-methyl-1-phenyl-1_H_-pyrazol-5-yl)thio)acetamide (7b)

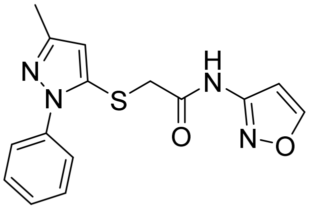

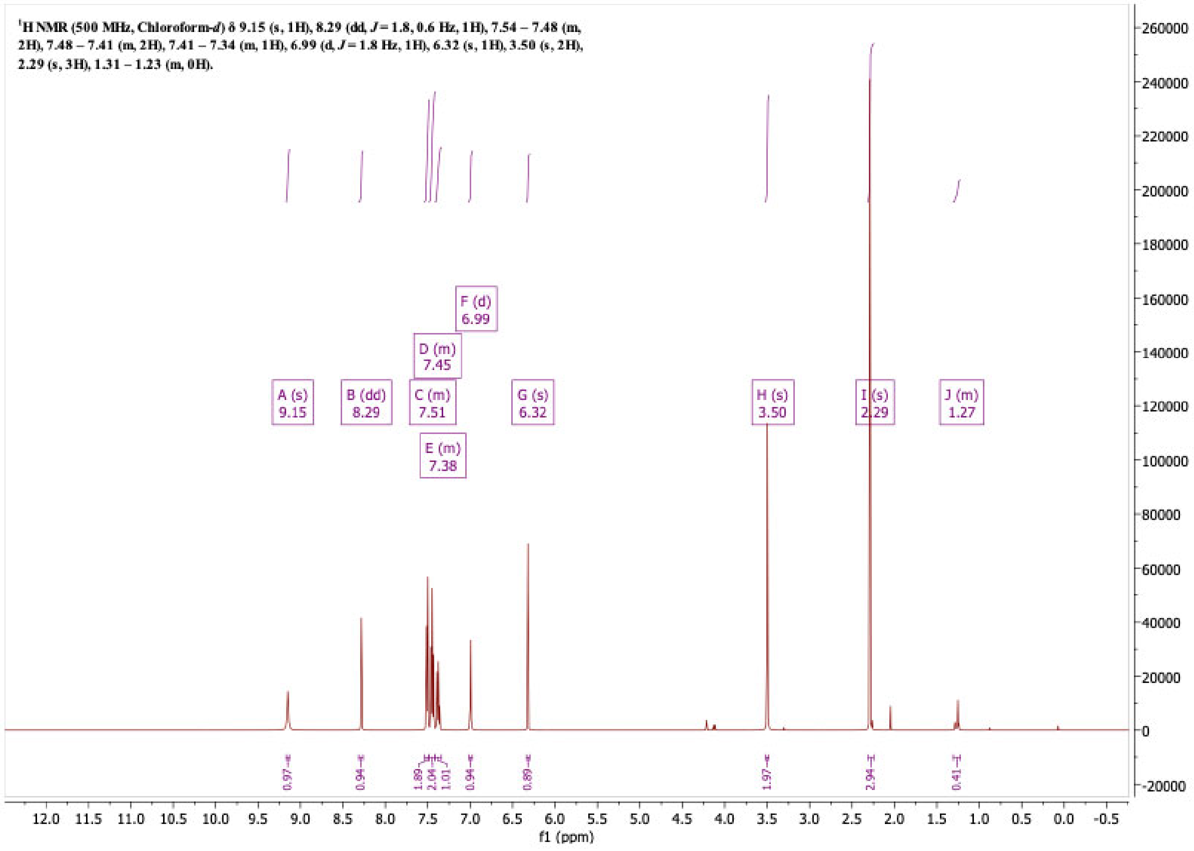

S3: ^{1}H NMR spectrum of _N_-(isoxazol-3-yl)-2-((3-methyl-1-phenyl-1_H_-pyrazol-5-yl)thio)acetamide (**7b**).

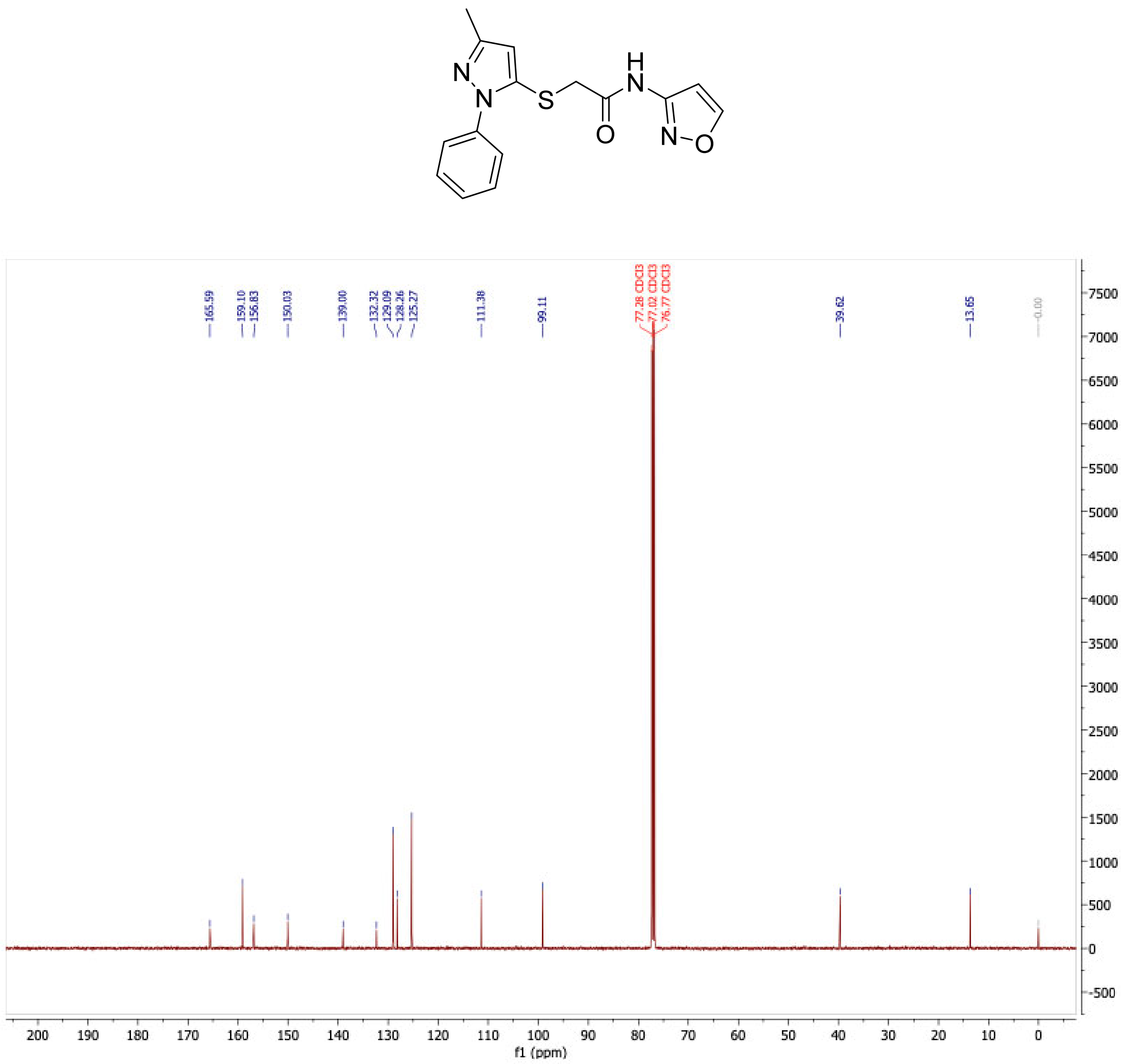

S4: ^{13}C NMR spectrum of *N*-(isoxazol-3-yl)-2-((3-methyl-1-phenyl-1*H*-pyrazol-5-yl)thio)acetamide (**7b**).

2-((3-methyl-1-phenyl-1*H*-pyrazol-5-yl)thio)-*N*-phenylacetamide (7c)

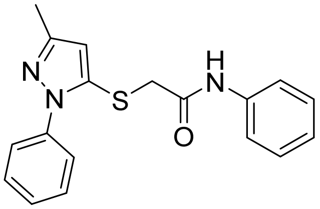

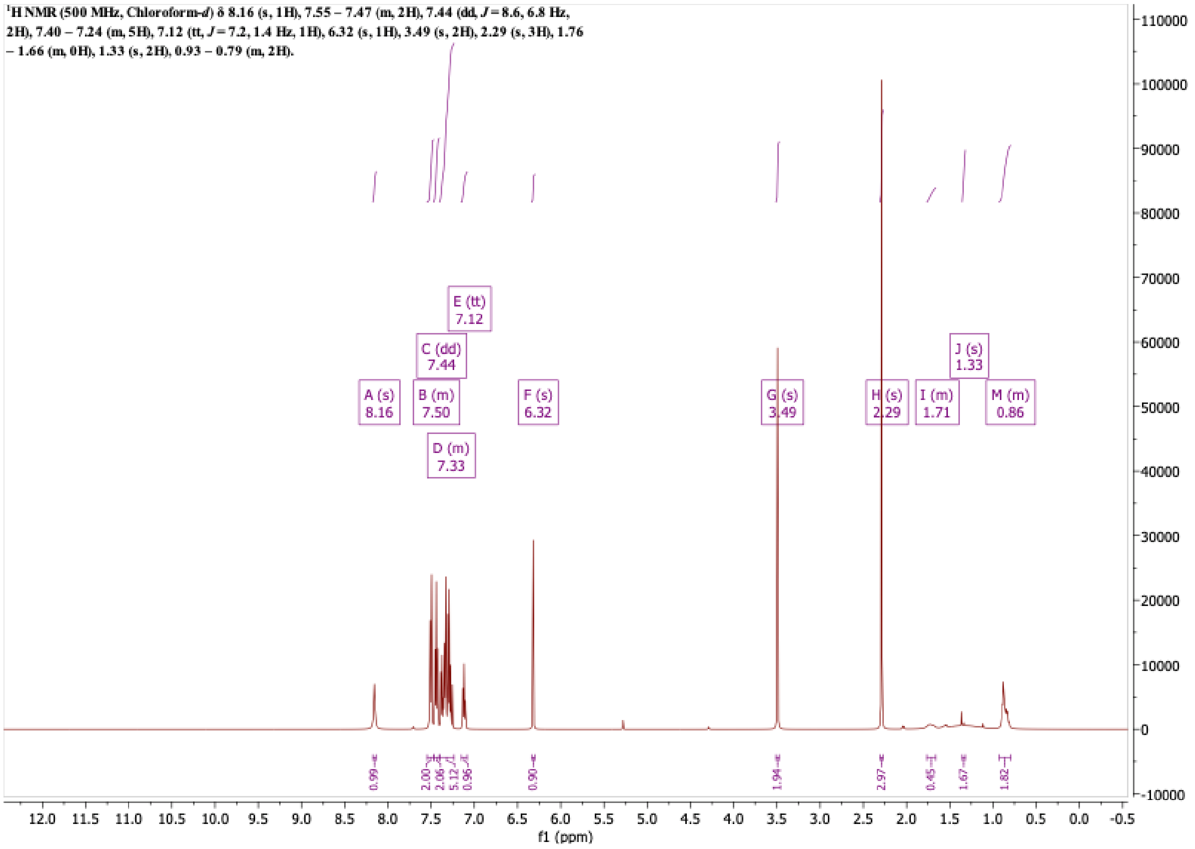

S5: ^{1}H NMR spectrum of 2-((3-methyl-1-phenyl-1*H*-pyrazol-5-yl)thio)-*N*-phenylacetamide (**7c**).

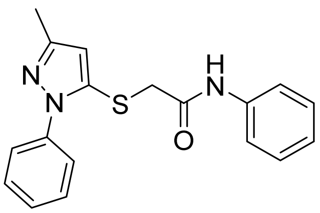

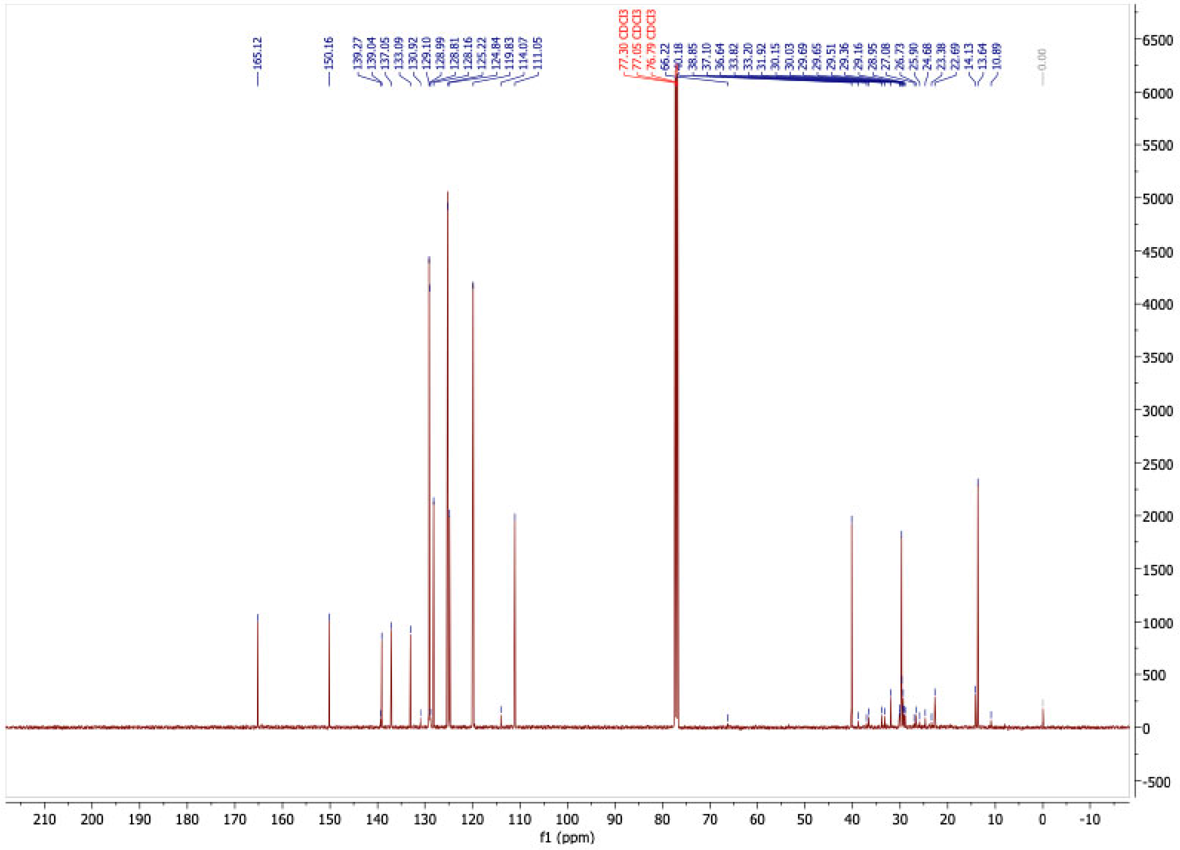

S6: ^{13}C NMR spectrum of 2-((3-methyl-1-phenyl-1*H*-pyrazol-5-yl)thio)-*N*-phenylacetamide (**7c**).

2-((3-methyl-1-phenyl-1*H*-pyrazol-5-yl)thio)-*N*-(pyridine-2-yl)acetamide (7d)

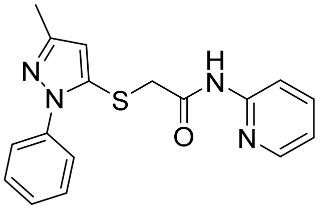

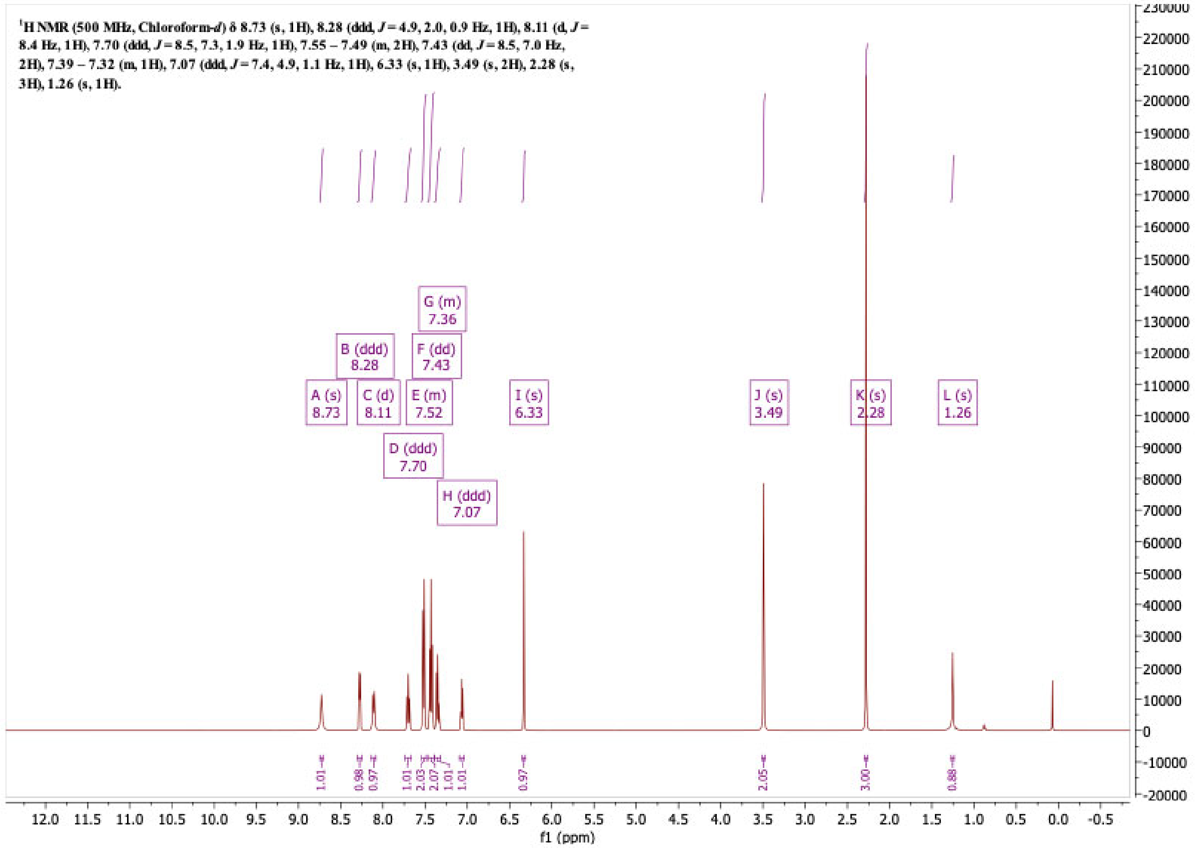

S7: ¹H NMR spectrum of 2-((3-methyl-1-phenyl-1*H*-pyrazol-5-yl)thio)-*N*-(pyridine-2-yl)acetamide (**7d**).

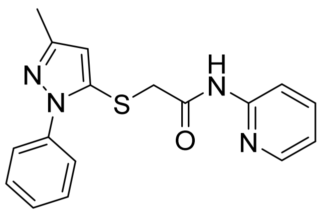

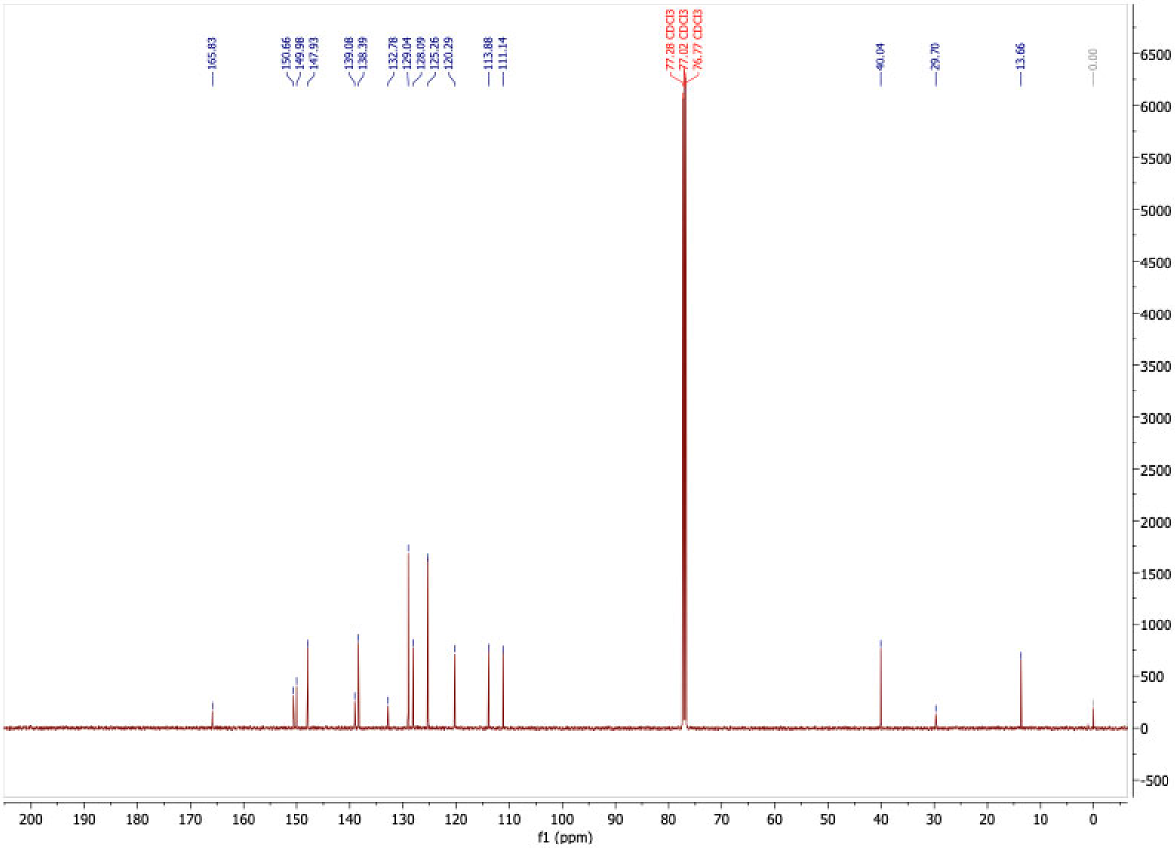

S8: ^{13}C NMR spectrum of 2-((3-methyl-1-phenyl-1*H*-pyrazol-5-yl)thio)-*N*-(pyridine-2-yl)acetamide (**7d**).

2-((3-methyl-1-phenyl-1*H*-pyrazol-5-yl)thio)-*N*-(5-methylpyridin-2-yl)acetamide (7e)

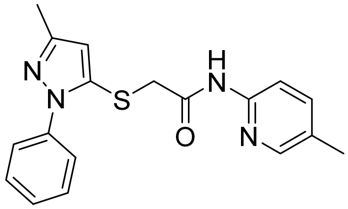

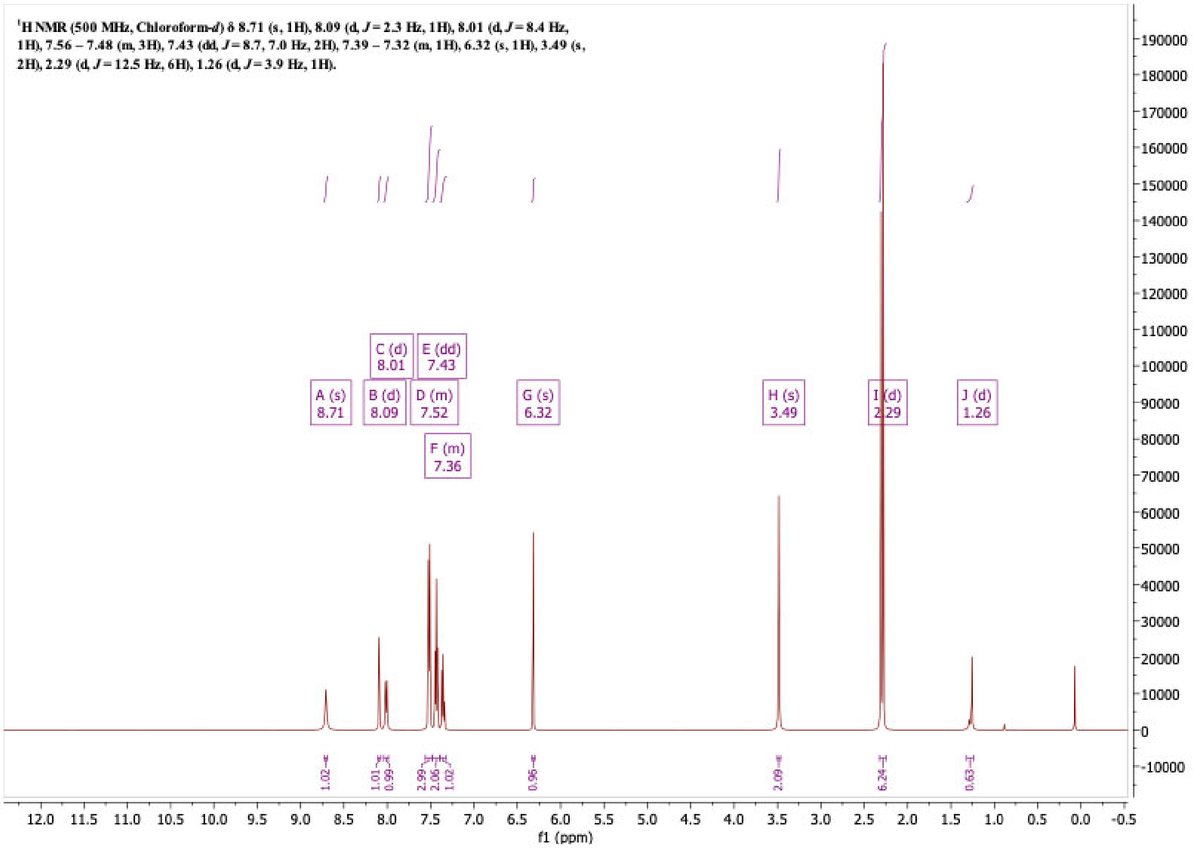

S9: ^{1}H NMR spectrum of 2-((3-methyl-1-phenyl-1*H*-pyrazol-5-yl)thio)-*N*-(5-methylpyridin-2-yl)acetamide (**7e**).

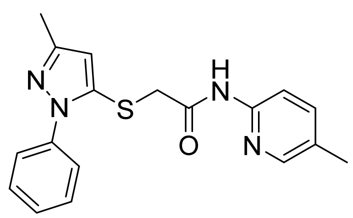

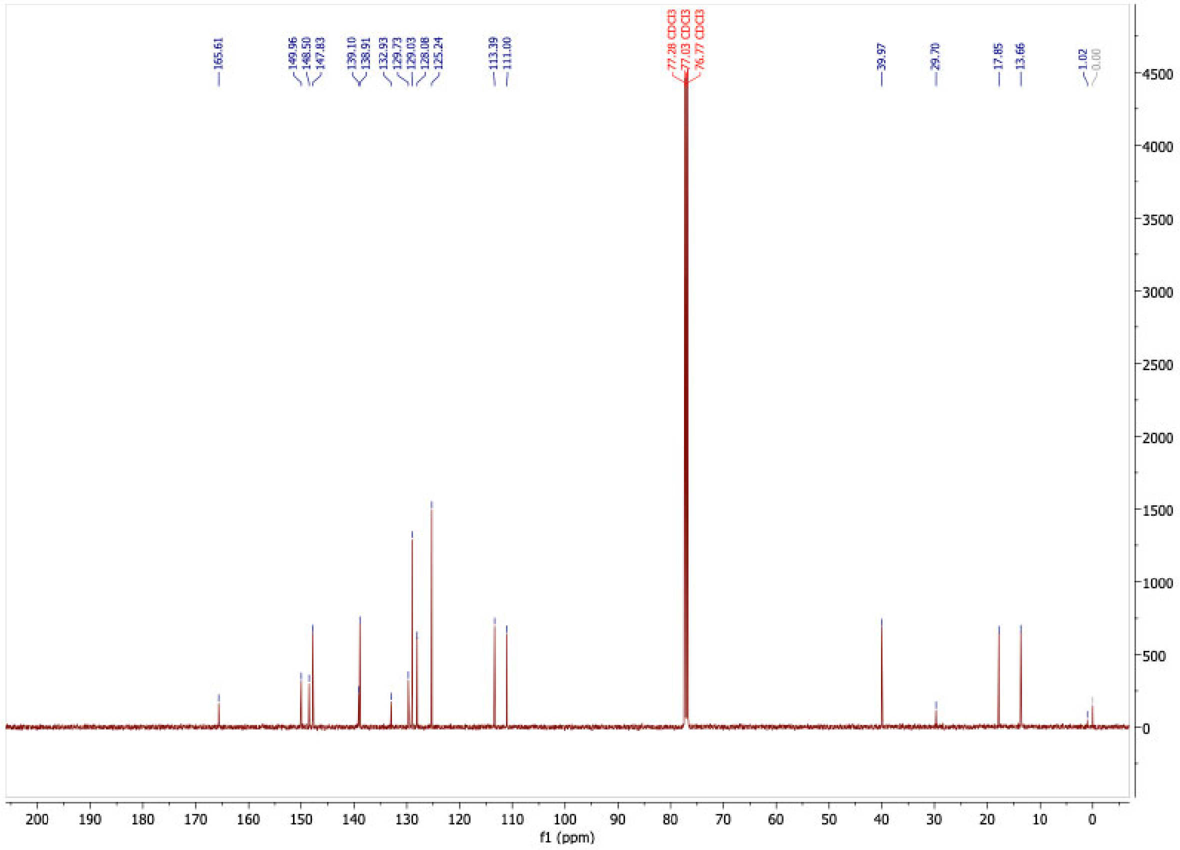

S10: ^{13}C NMR spectrum of 2-((3-methyl-1-phenyl-1*H*-pyrazol-5-yl)thio)-*N*-(5-methylpyridin-2-yl)acetamide (**7e**).

N-(5-chloropyridin-2-yl)-2-((3-methyl-1-phenyl-1H-pyrazol-5-yl)thio)acetamide (7f)

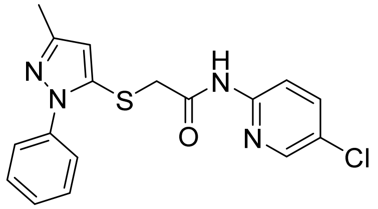

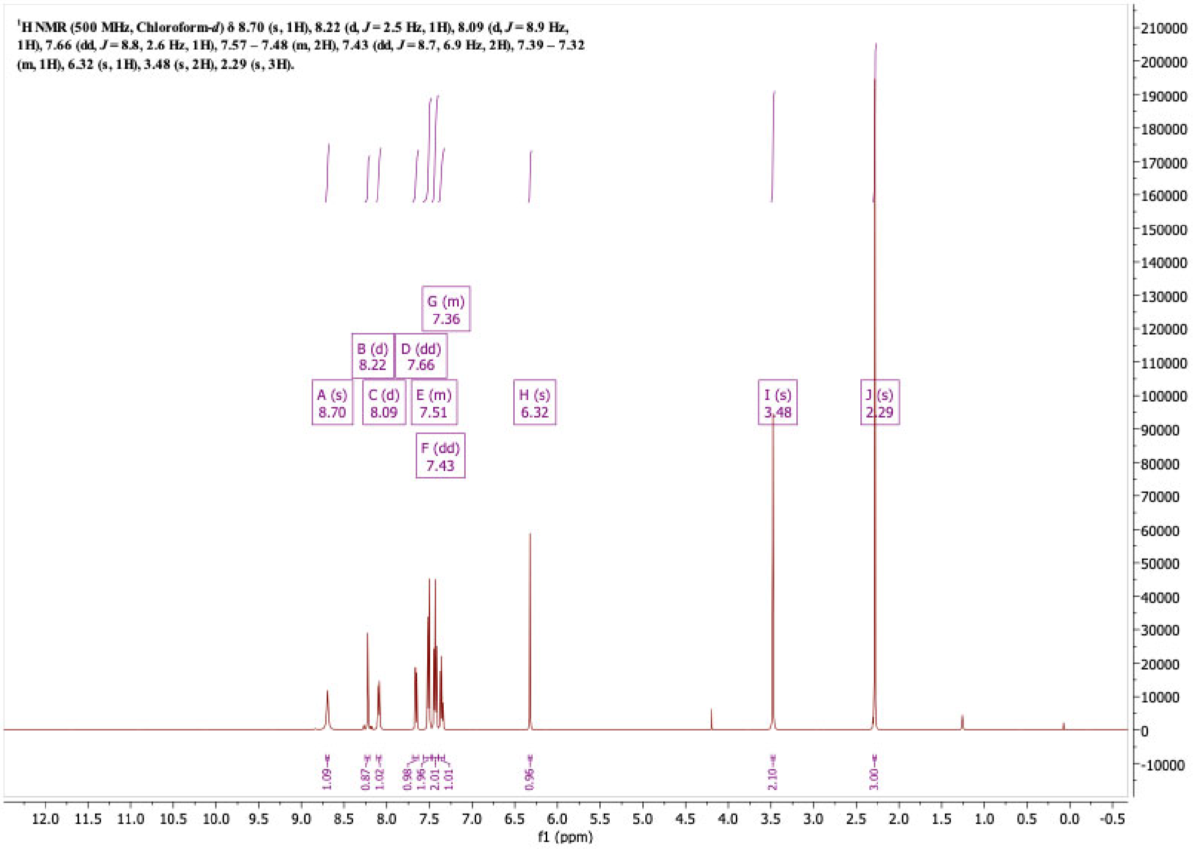

S11: ^{1}H NMR spectrum of N-(5-chloropyridin-2-yl)-2-((3-methyl-1-phenyl-1H-pyrazol-5-yl)thio)acetamide (**7f**).

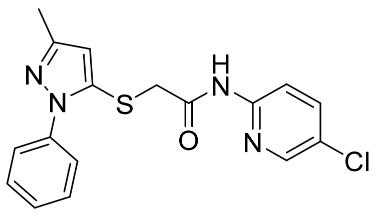

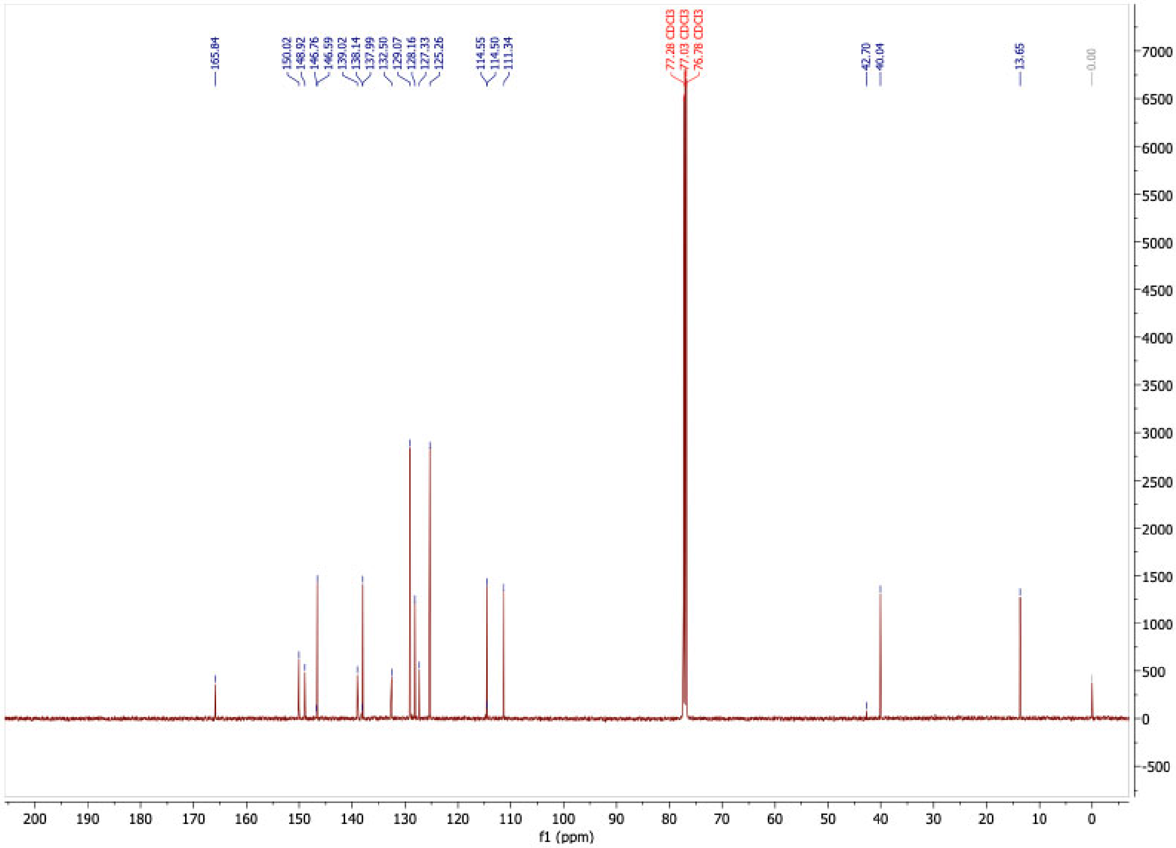

S12: ^{13}C NMR spectrum of *N*-(5-chloropyridin-2-yl)-2-((3-methyl-1-phenyl-1*H*-pyrazol-5-yl)thio)acetamide (**7f**).

2-((3-methyl-1-phenyl-1*H*-pyrazol-5-yl)thio)-*N*-(pyrimidin-2-yl)acetamide (7g)

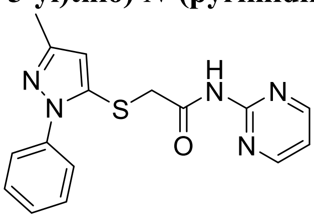

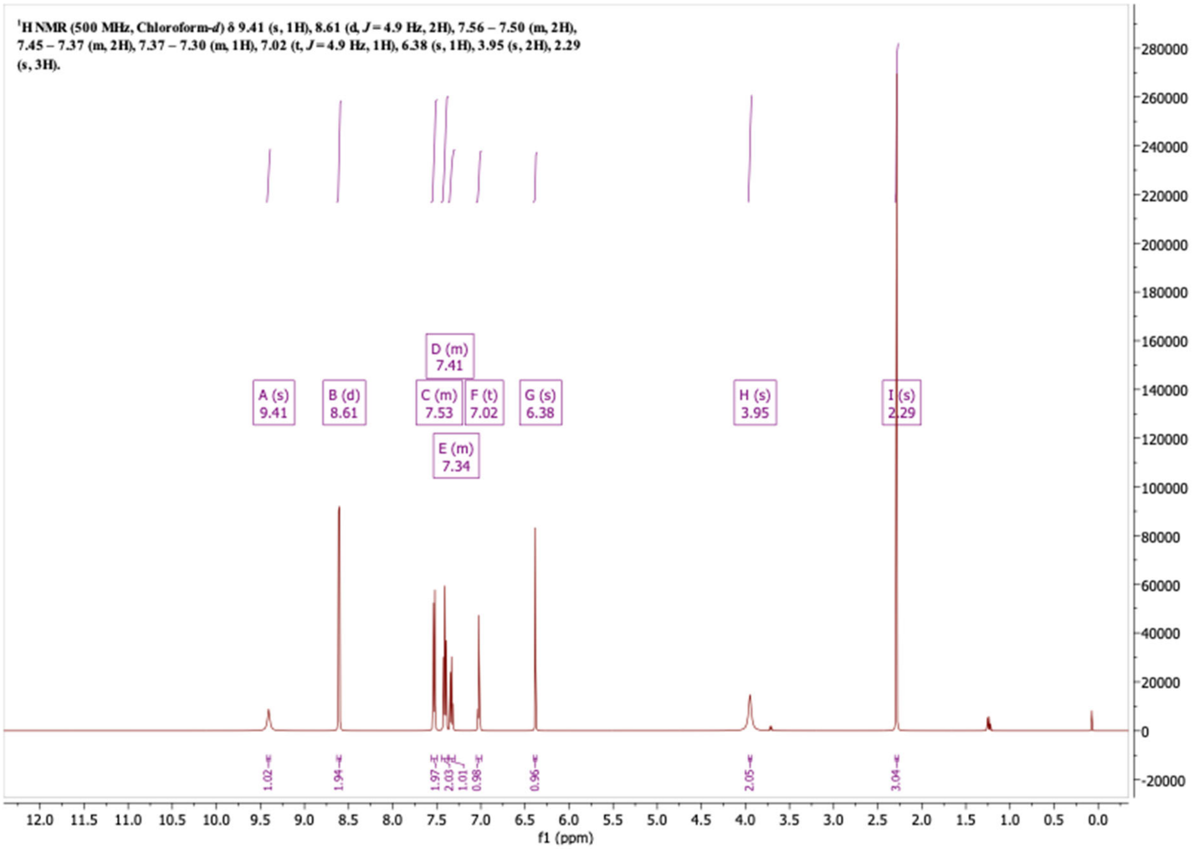

S13: ^{1}H NMR spectrum of 2-((3-methyl-1-phenyl-1*H*-pyrazol-5-yl)thio)-*N*-(pyrimidin-2-yl)acetamide (**7g**).

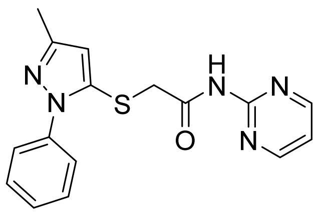

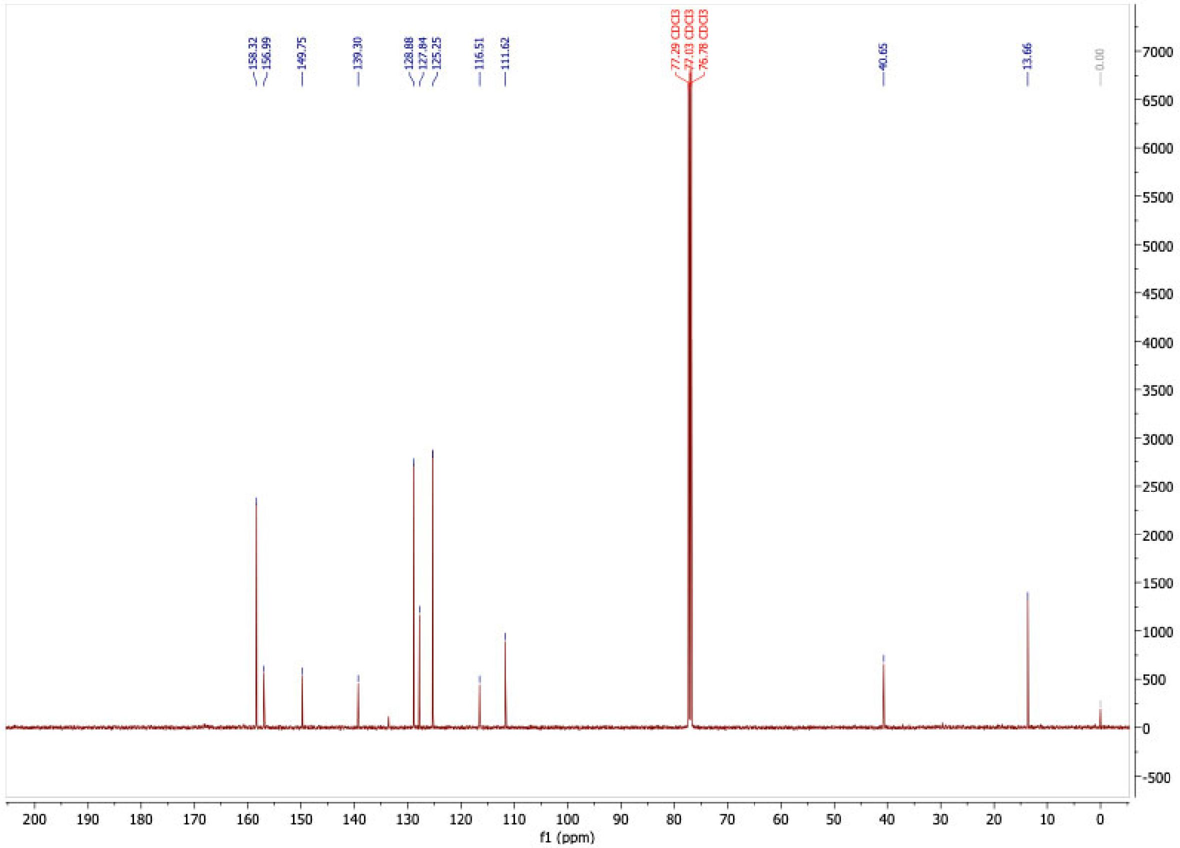

S14: ^{13}C NMR spectrum of 2-((3-methyl-1-phenyl-1*H*-pyrazol-5-yl)thio)-*N*-(pyrimidin-2-yl)acetamide (**7g**).

2-((3-methyl-1-phenyl-1H-pyrazol-5-yl)thio)-N-(pyrazin-2-yl)acetamide (7h)

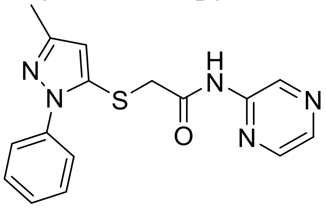

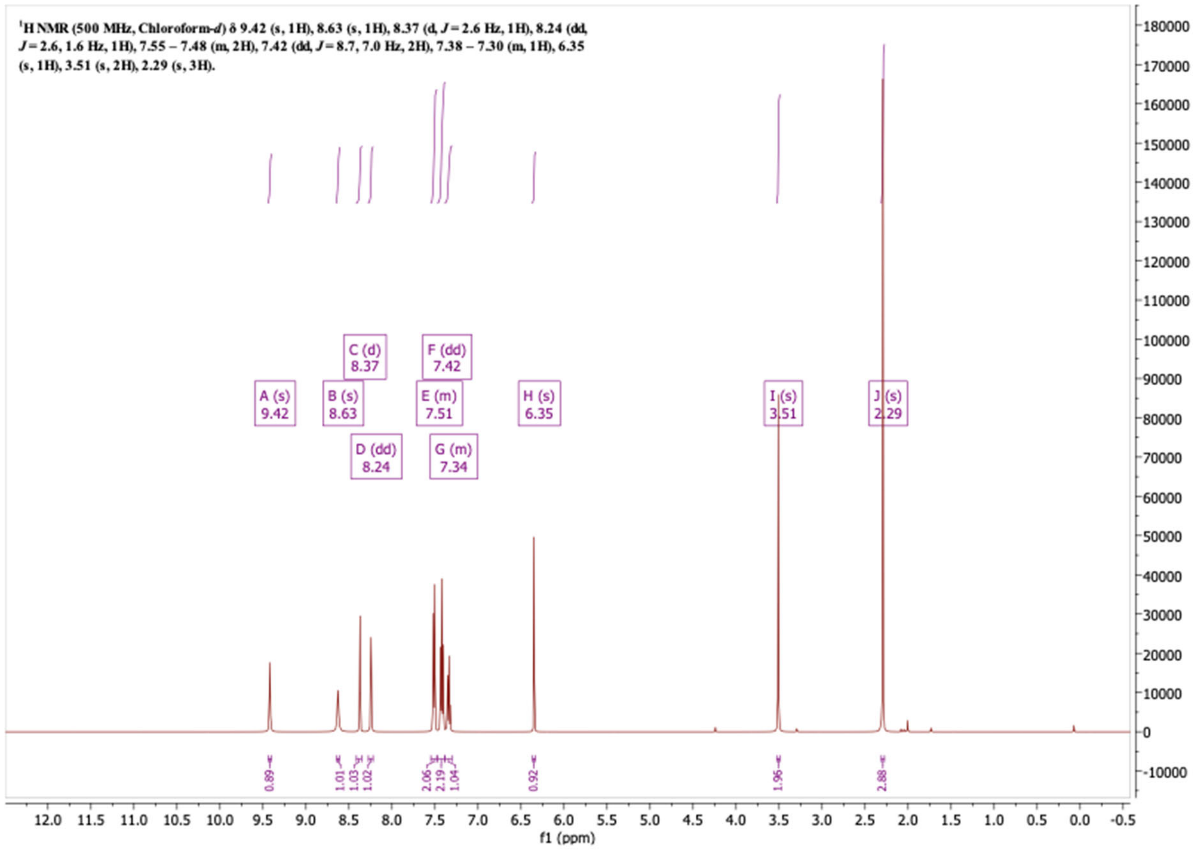

S15: ^{1}H NMR spectrum of 2-((3-methyl-1-phenyl-1H-pyrazol-5-yl)thio)-N-(pyrazin-2-yl)acetamide (**7h**).

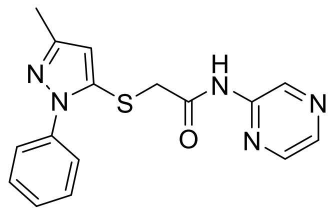

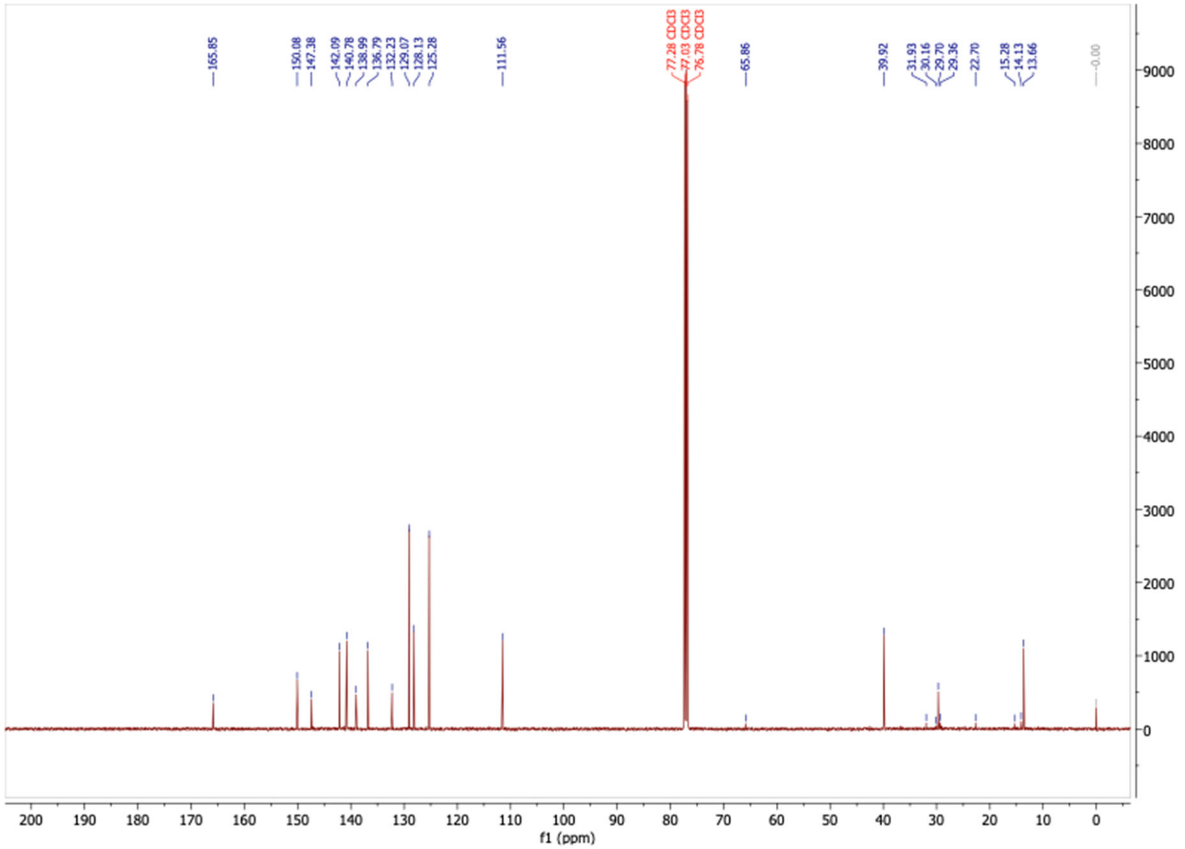

S16: ^{13}C NMR spectrum of 2-((3-methyl-1-phenyl-1H-pyrazol-5-yl)thio)-N-(pyrazin-2-yl)acetamide (**7h**).

N-(6-chloropyrazin-2-yl)-2-((3-methyl-1-phenyl-1H-pyrazol-5-yl)thio)acetamide (7i)

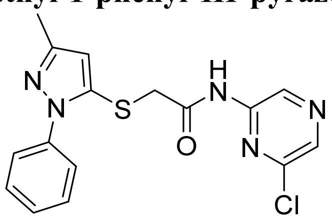

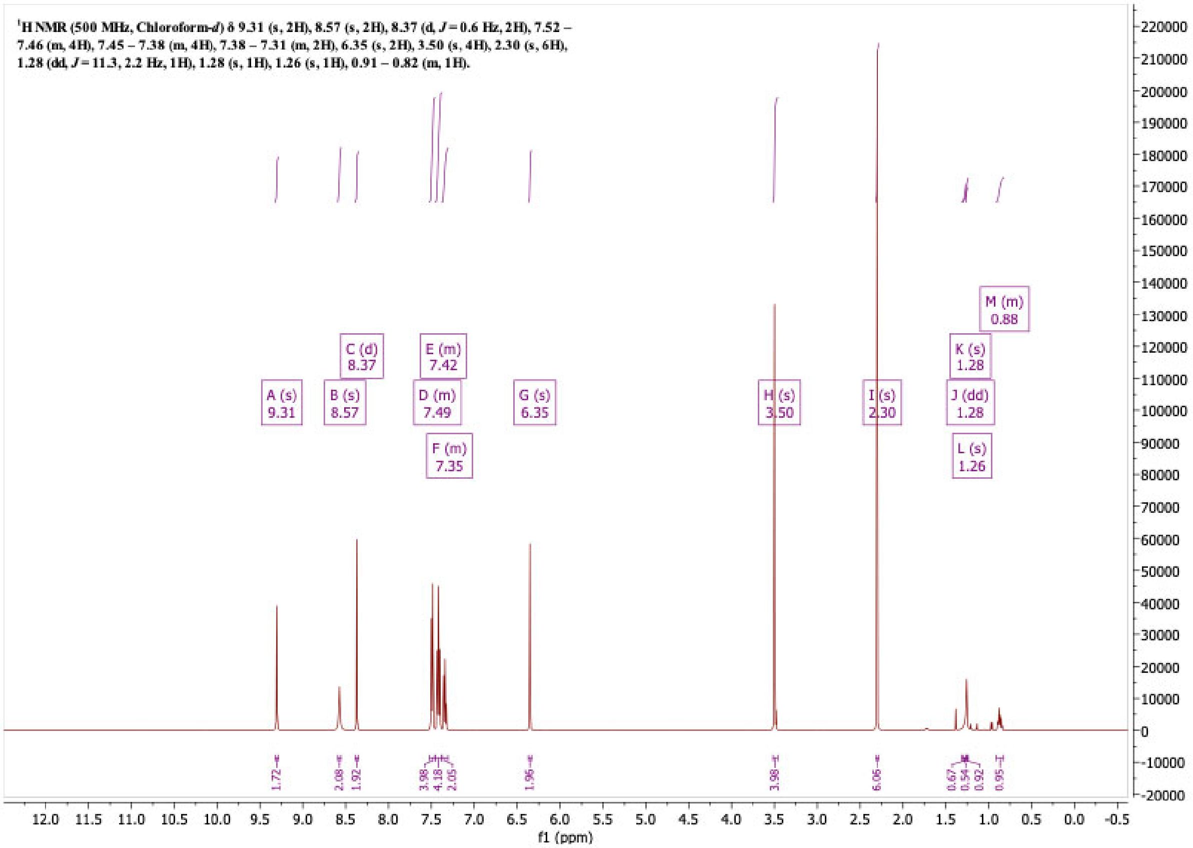

S17: ^{1}H NMR spectrum of *N*-(6-chloropyrazin-2-yl)-2-((3-methyl-1-phenyl-1*H*-pyrazol-5-yl)thio)acetamide (**7i**).

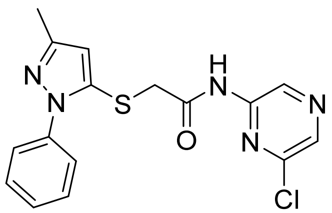

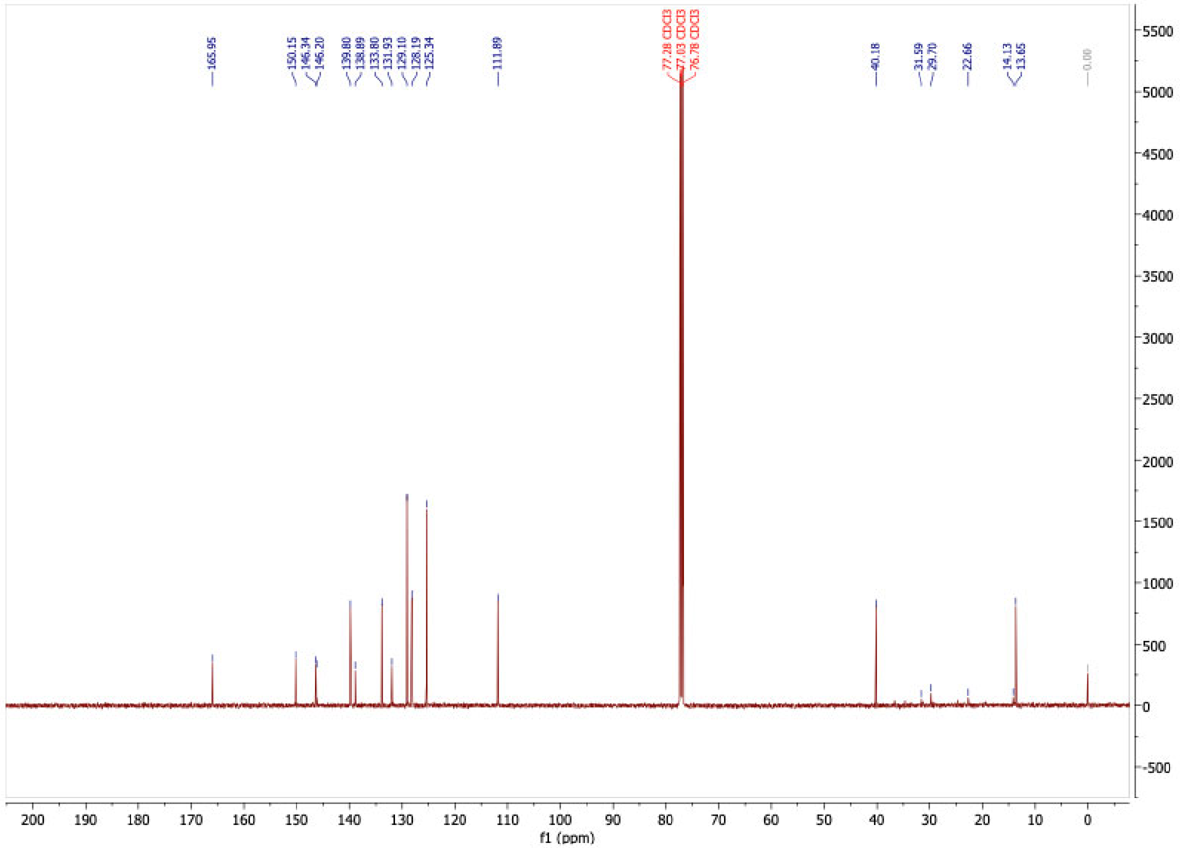

S18: ^{13}C NMR spectrum of *N*-(6-chloropyrazin-2-yl)-2-((3-methyl-1-phenyl-1*H*-pyrazol-5-yl)thio)acetamide (**7i**).

N,N-diethyl-2-((3-methyl-1-phenyl-1H-pyrazol-5-yl)thio)acetamide (7j)

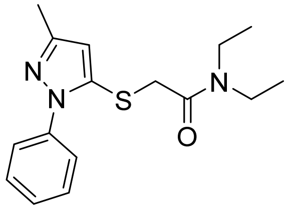

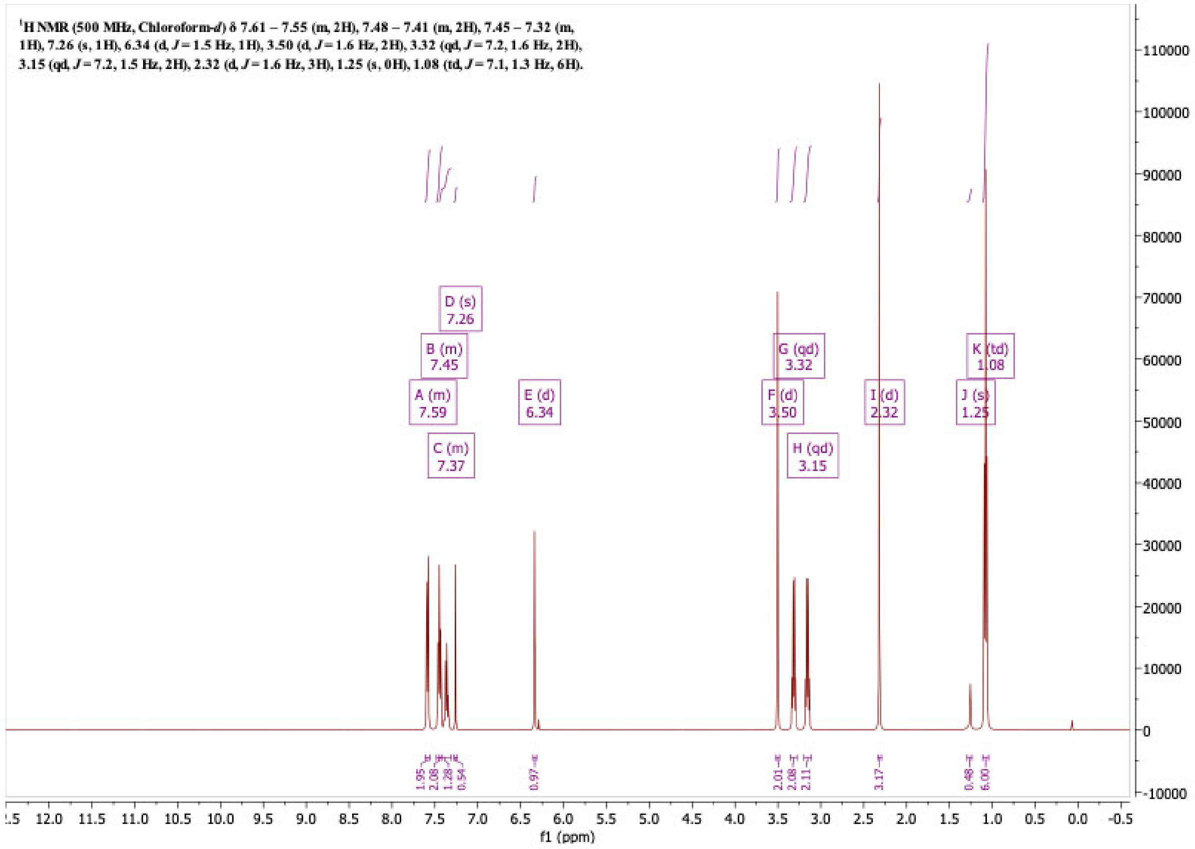

S19: ^{1}H NMR spectrum of N,N-diethyl-2-((3-methyl-1-phenyl-1H-pyrazol-5-yl)thio)acetamide (**7j**).

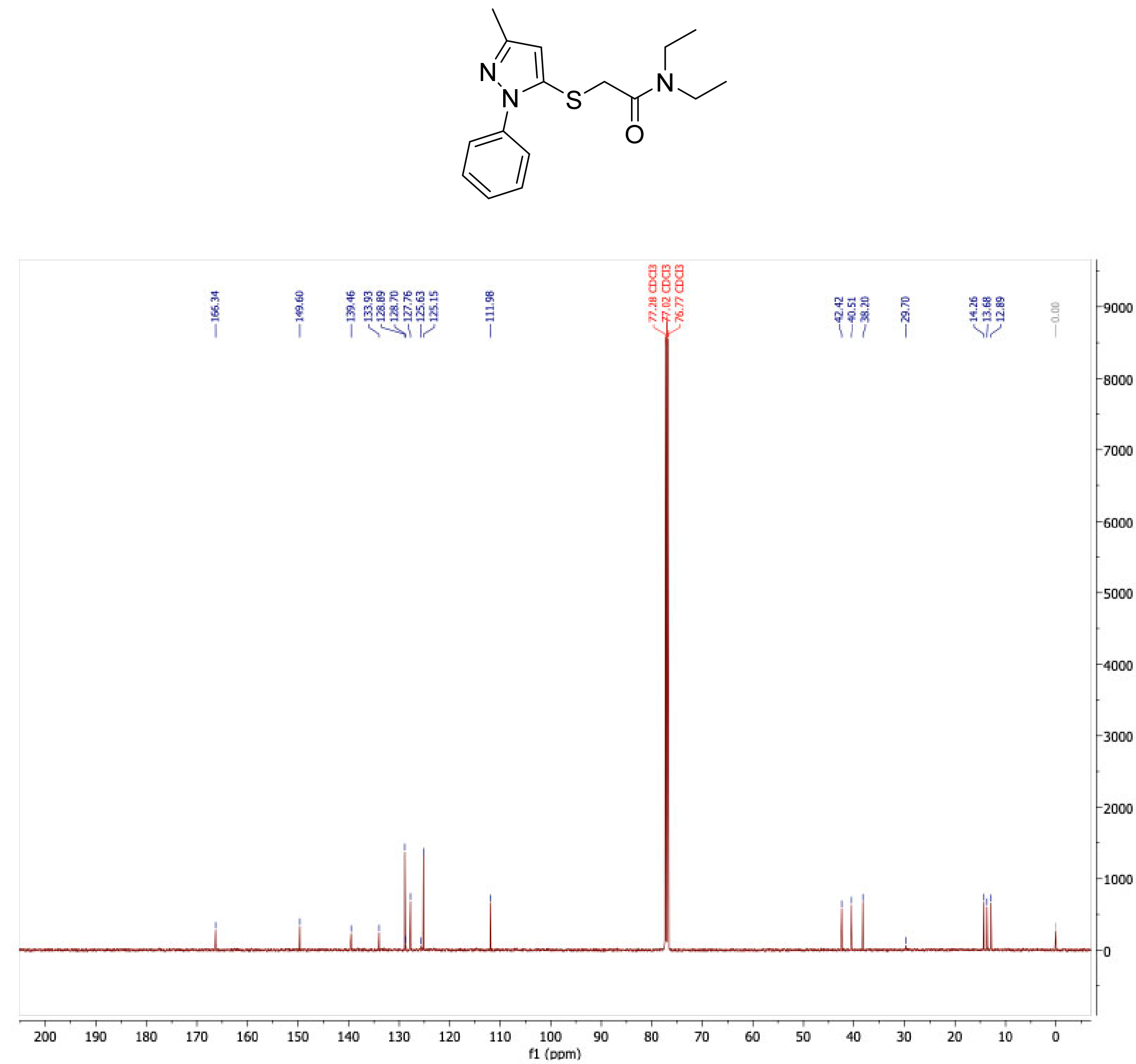

S20: ^{13}C NMR spectrum of *N*,N-diethyl-2-((3-methyl-1-phenyl-1*H*-pyrazol-5-yl)thio)acetamide (**7j**).

***N*-(1*H*-benzo[d]imidazol-2-yl)-2-((3-methyl-1-phenyl-1H-pyrazol-5-yl)thio)acetamide (7k)**

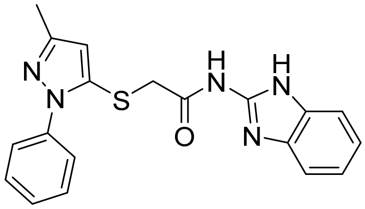

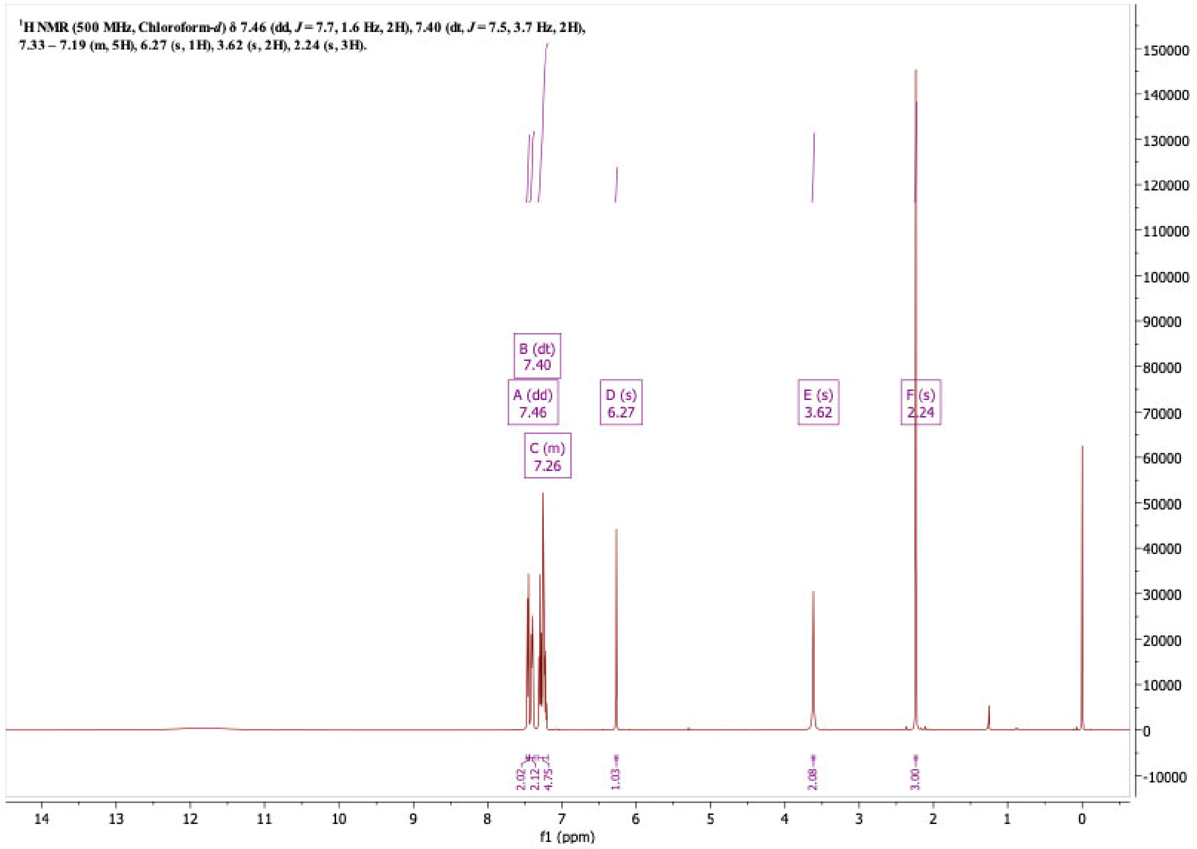

S21: ^{1}H NMR spectrum of *N*-(1*H*-benzo[d]imidazol-2-yl)-2-((3-methyl-1-phenyl-1H-pyrazol-5-yl)thio)acetamide (**7k**).

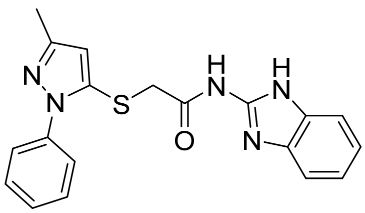

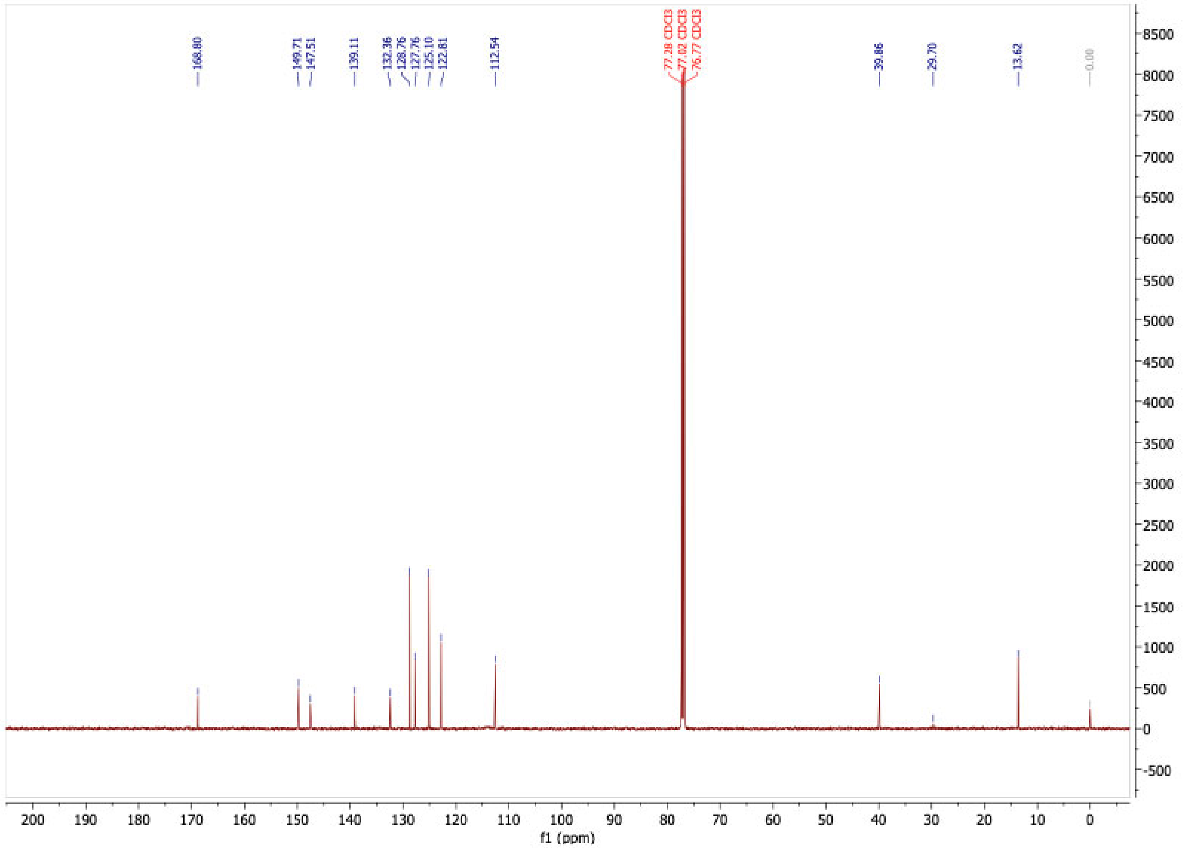

S22: ^{13}C NMR spectrum of *N*-(1*H*-benzo[d]imidazol-2-yl)-2-((3-methyl-1-phenyl-1H-pyrazol-5-yl)thio)acetamide (**7k**).

2-((3-methyl-1-phenyl-1*H*-pyrazol-5-yl)thio)-*N*-(thiazol-2-yl)propanamide (7l)

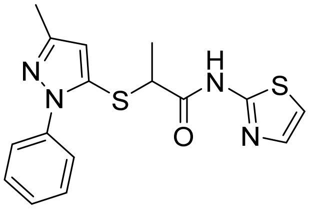

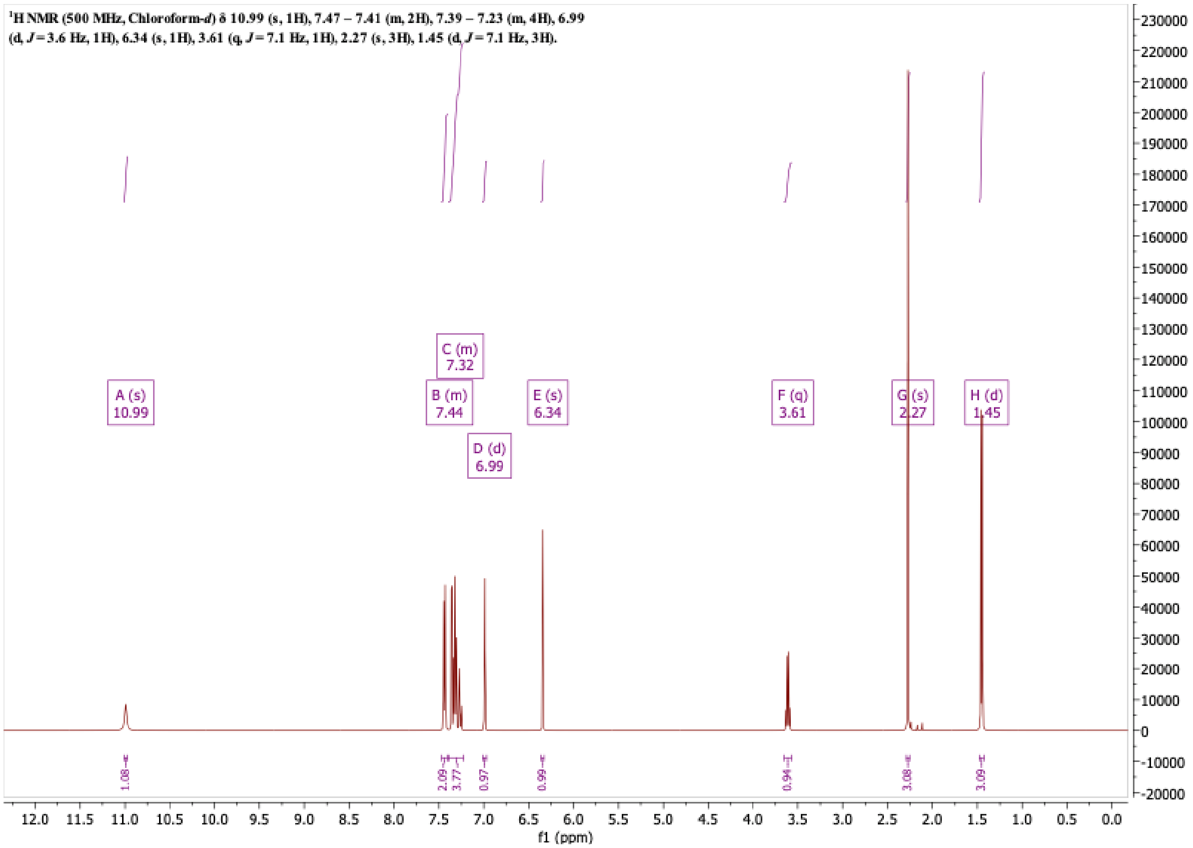

S23: ¹H NMR spectrum of 2-((3-methyl-1-phenyl-1*H*-pyrazol-5-yl)thio)-*N*-(thiazol-2-yl)propanamide (**7l**).

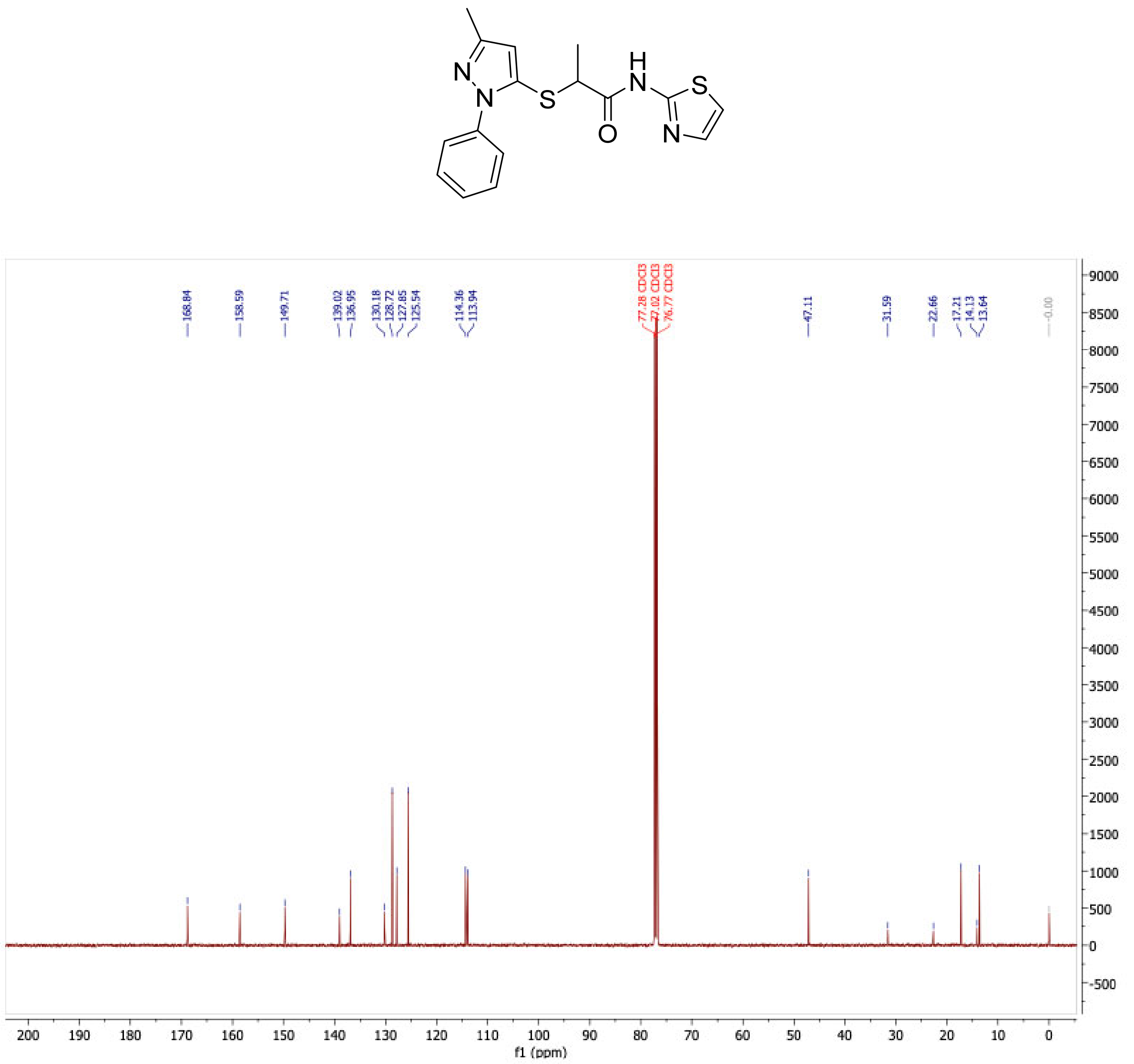

S24: ^{13}C NMR spectrum of 2-((3-methyl-1-phenyl-1*H*-pyrazol-5-yl)thio)-*N*-(thiazol-2-yl)propanamide (**7l**).

N-(isoxazol-3-yl)-2-((3-methyl-1-phenyl-1H-pyrazol-5-yl)thio)propanamide (7m)

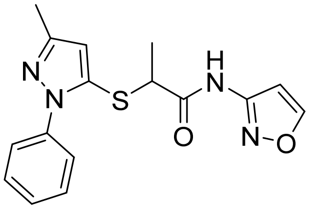

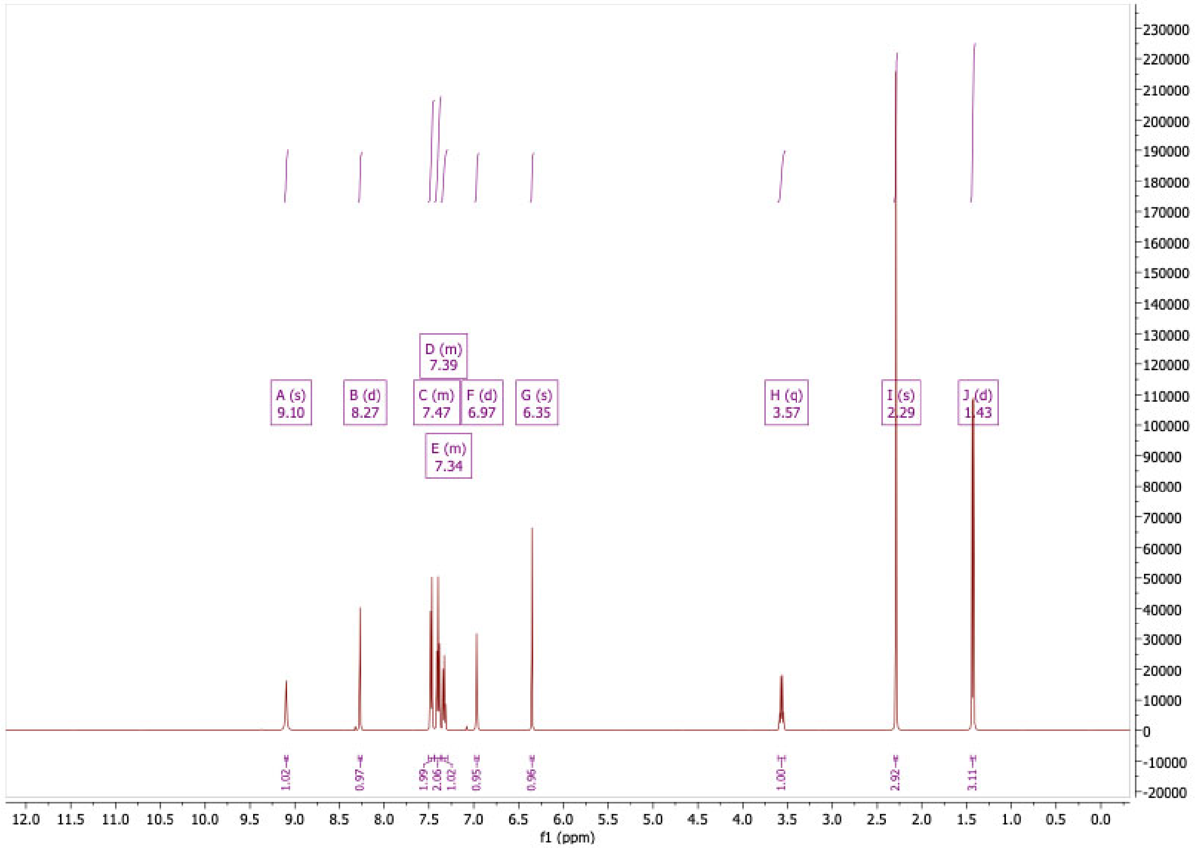

S25: ^{1}H NMR spectrum of *N*-(isoxazol-3-yl)-2-((3-methyl-1-phenyl-1*H*-pyrazol-5-yl)thio)propanamide (**7m**).

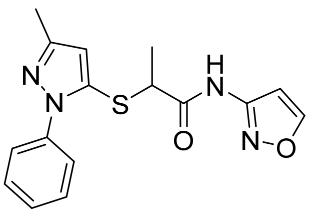

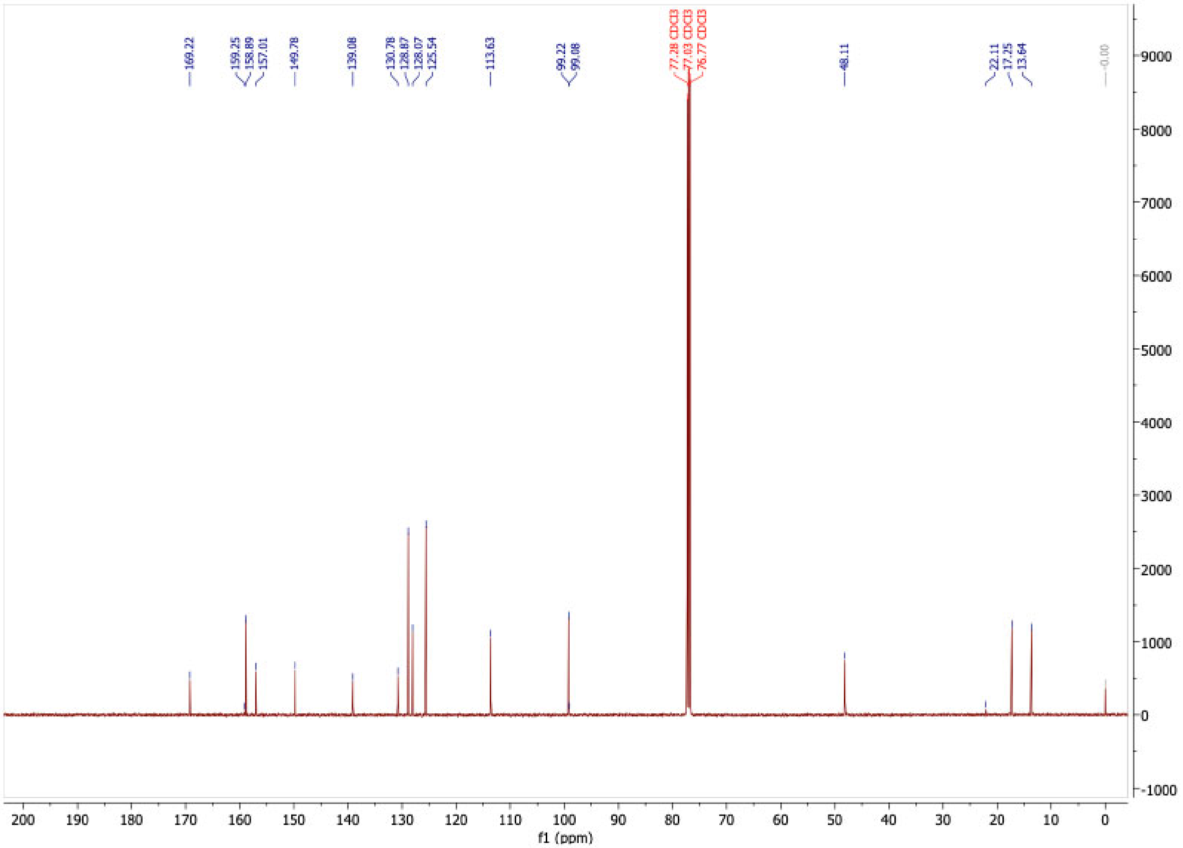

S26: ^{13}C NMR spectrum of *N*-(isoxazol-3-yl)-2-((3-methyl-1-phenyl-1*H*-pyrazol-5-yl)thio)propanamide (**7m**).

2-((3-methyl-1-phenyl-1*H*-pyrazol-5-yl)thio)-*N*-phenylpropanamide (7n)

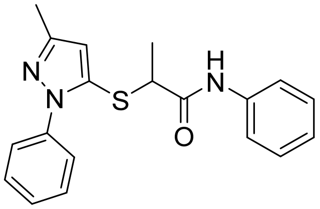

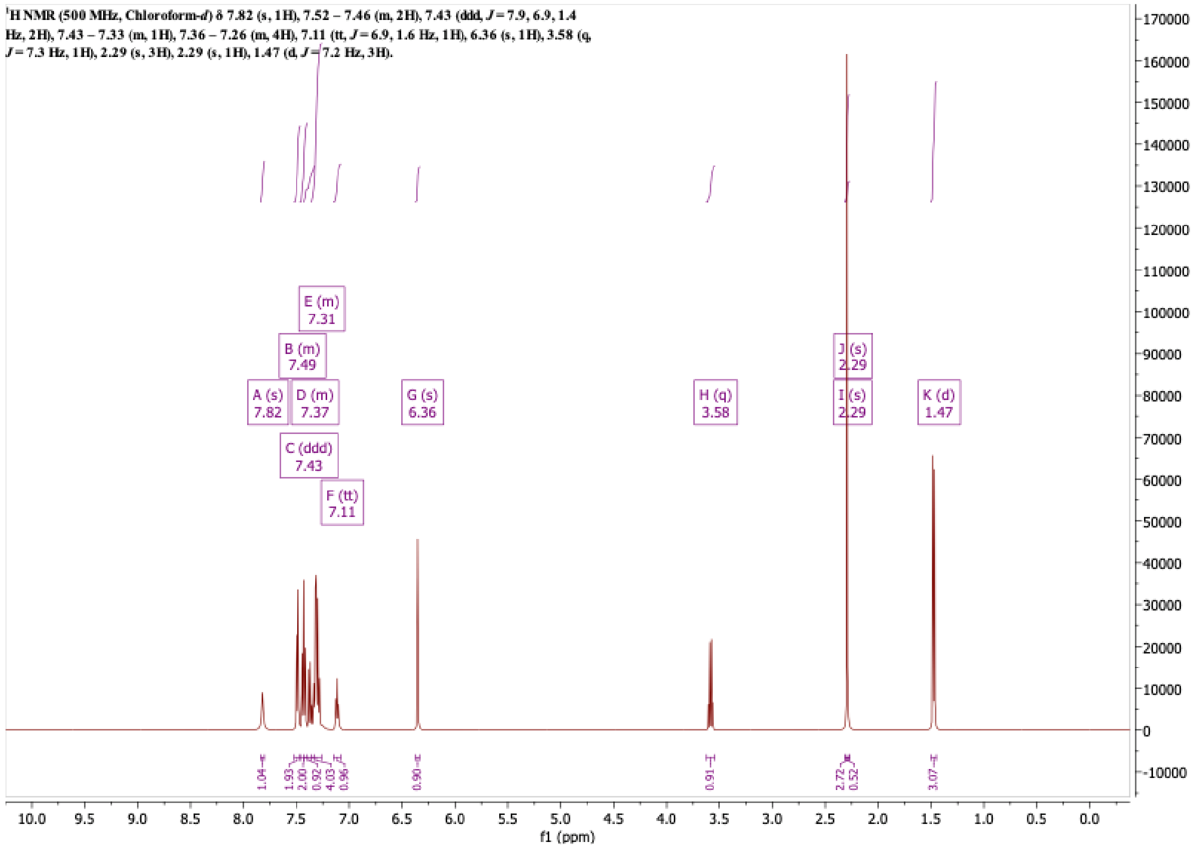

S27: ^{1}H NMR spectrum of 2-((3-methyl-1-phenyl-1*H*-pyrazol-5-yl)thio)-*N*-phenylpropanamide (**7n**).

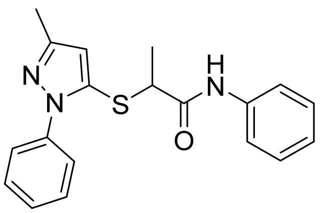

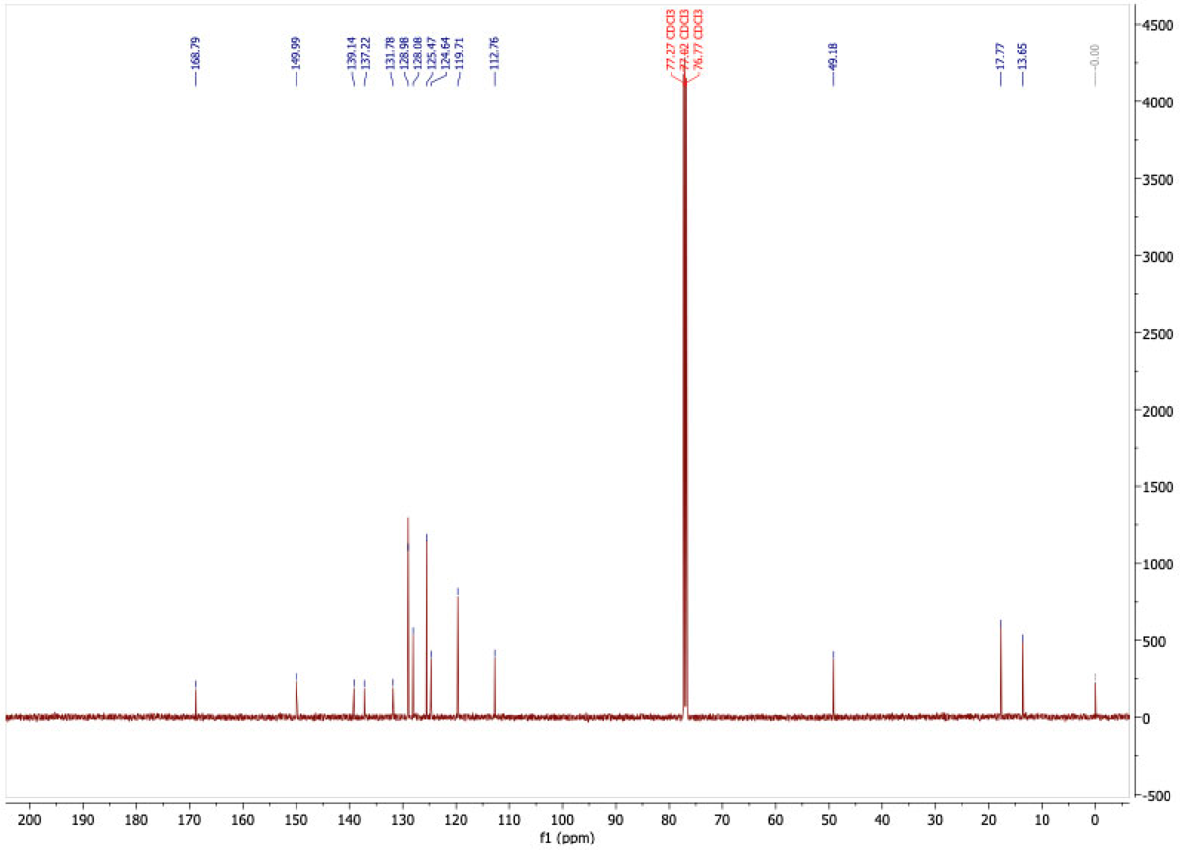

S28: ^{13}C NMR spectrum of 2-((3-methyl-1-phenyl-1*H*-pyrazol-5-yl)thio)-*N*-phenylpropanamide (**7n**).

N-benzyl-2-((3-methyl-1-phenyl-1*H*-pyrazol-5-yl)thio)propanamide (7o)

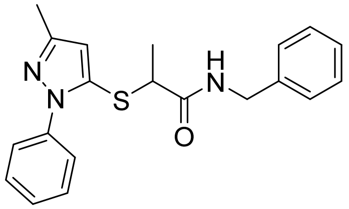

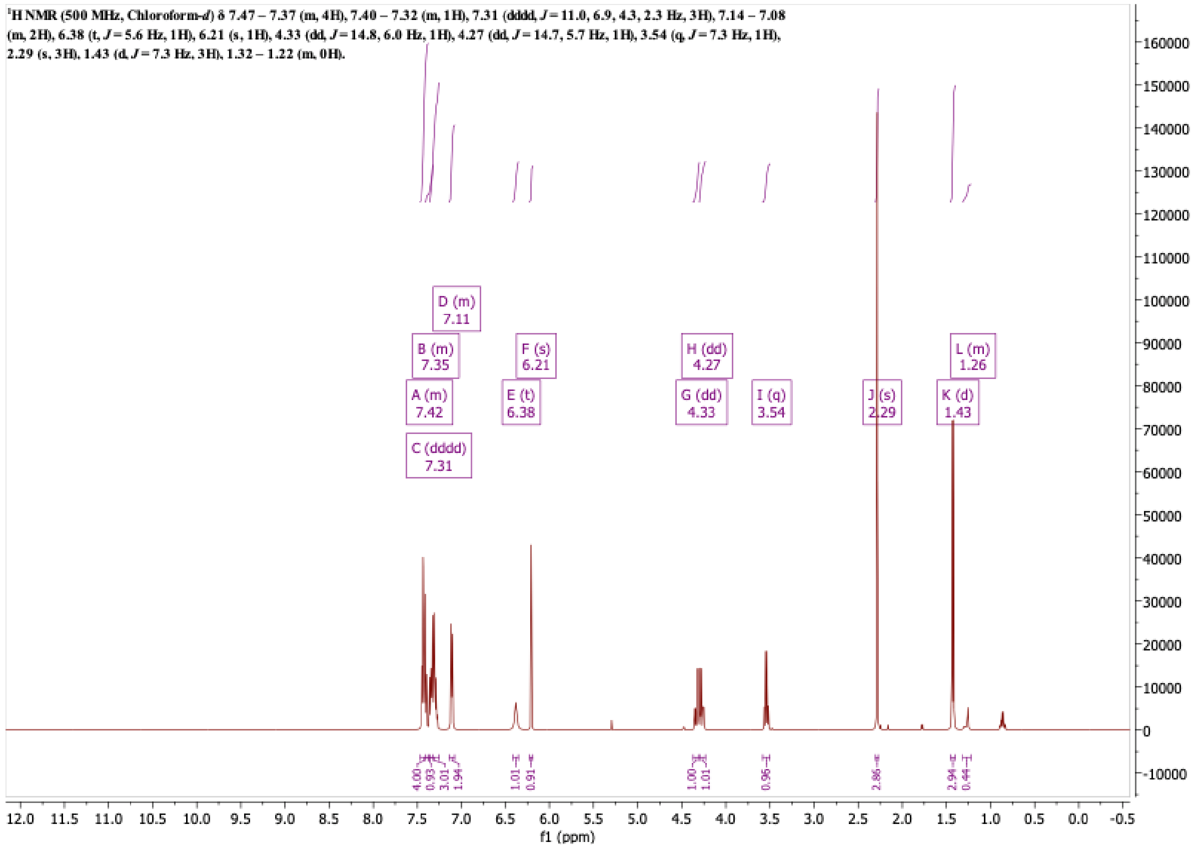

S29: ^{1}H NMR spectrum of *N*-benzyl-2-((3-methyl-1-phenyl-1*H*-pyrazol-5-yl)thio)propanamide (**7o**).

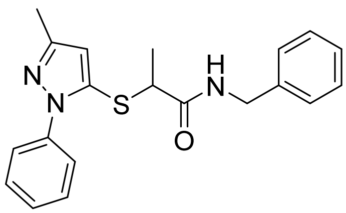

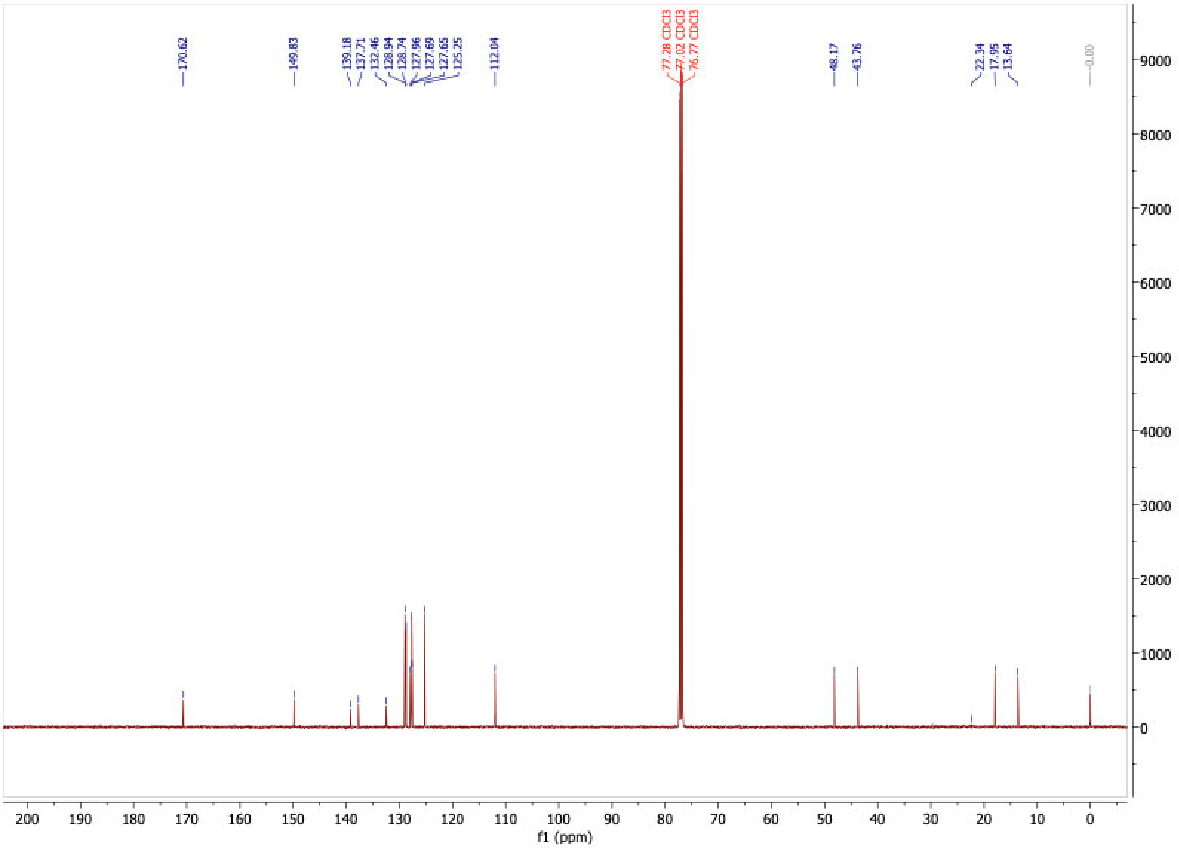

S30: ^{13}C NMR spectrum of *N*-benzyl-2-((3-methyl-1-phenyl-1*H*-pyrazol-5-yl)thio)propanamide (**7o**).

2-((3-methyl-1-phenyl-1*H*-pyrazol-5-yl)thio)-*N*-(pyridine-2-yl)propanamide (7p)

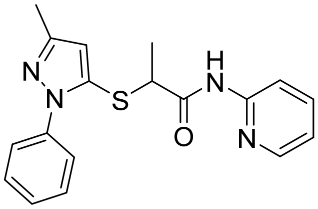

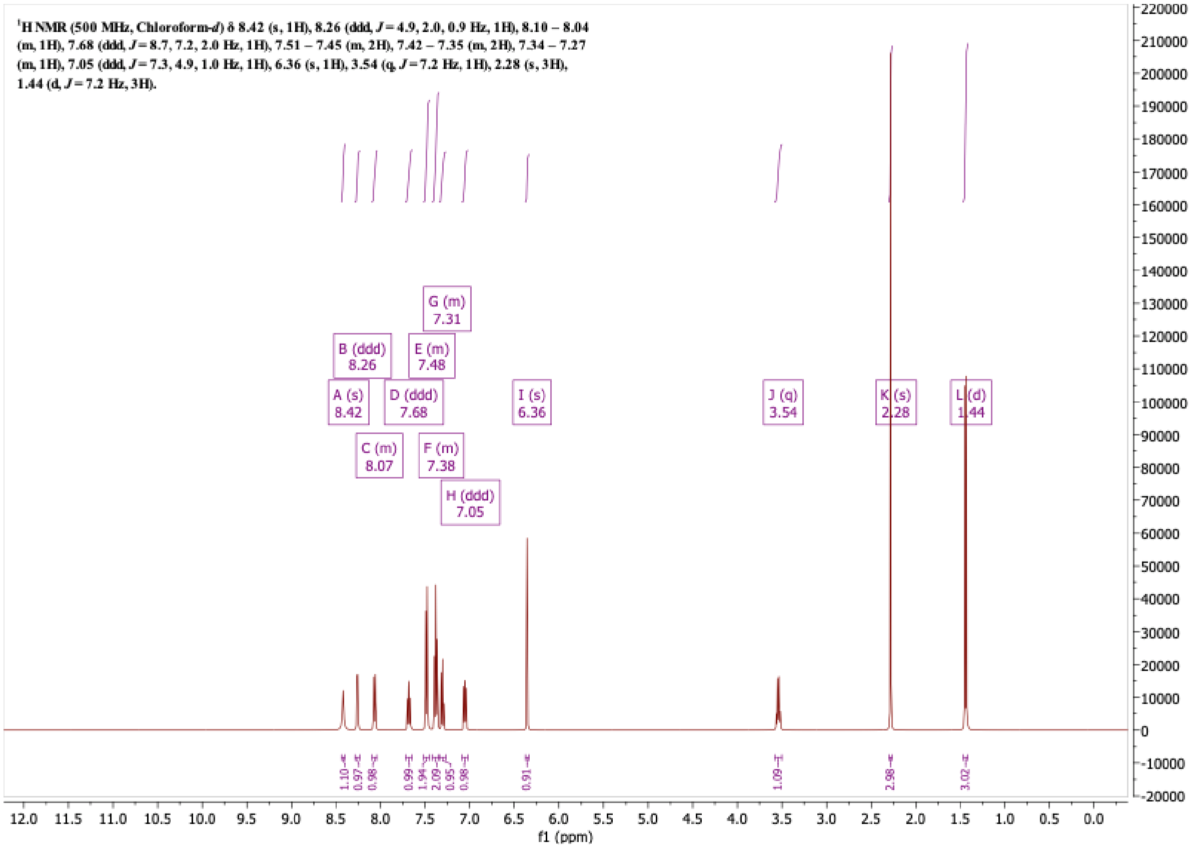

S31: ^{1}H NMR spectrum of 2-((3-methyl-1-phenyl-1*H*-pyrazol-5-yl)thio)-*N*-(pyridine-2-yl)propanamide (**7p**).

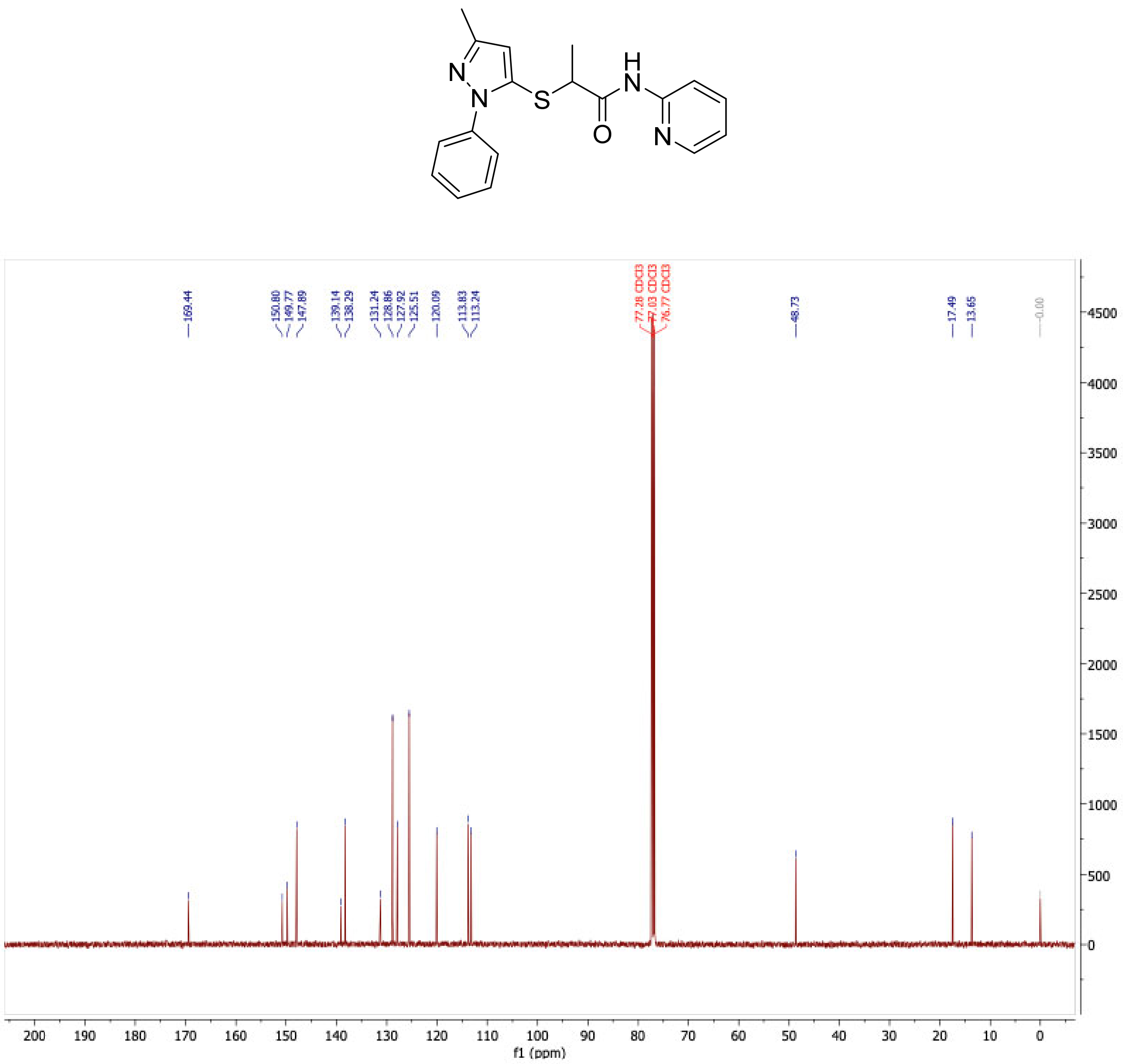

S32: ^{13}C NMR spectrum of 2-((3-methyl-1-phenyl-1*H*-pyrazol-5-yl)thio)-*N*-(pyridine-2-yl)propanamide (**7p**).

(*R*)-2-((3-methyl-1-phenyl-1*H*-pyrazol-5-yl)thio)-*N*-(pyridin-2-yl)propenamide (7q)

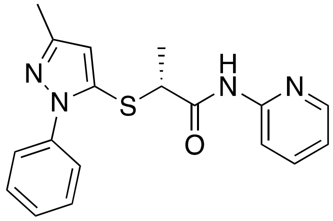

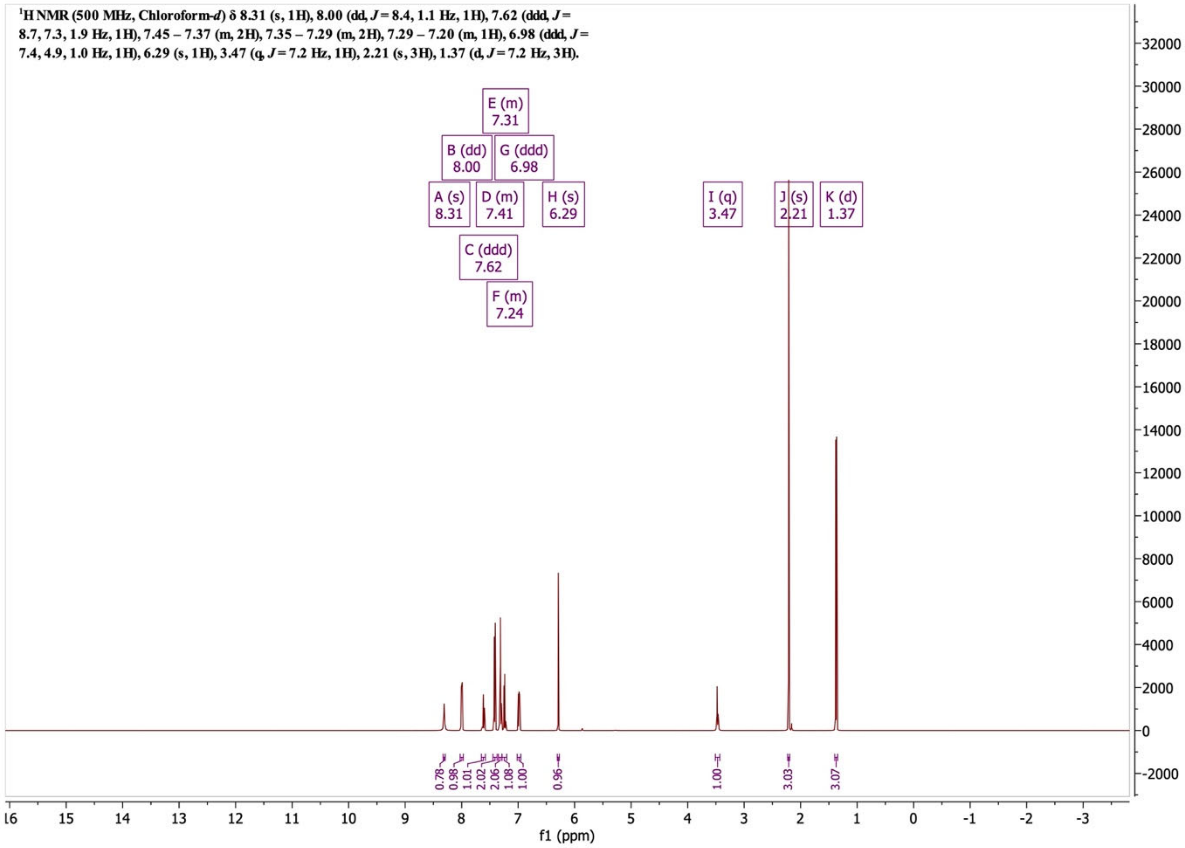

S33: ^{1}H NMR spectrum of (*R*)-2-((3-methyl-1-phenyl-1*H*-pyrazol-5-yl)thio)-*N*-(pyridin-2-yl)propenamide (**7q**).

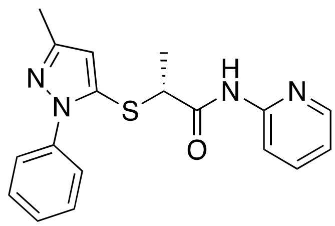

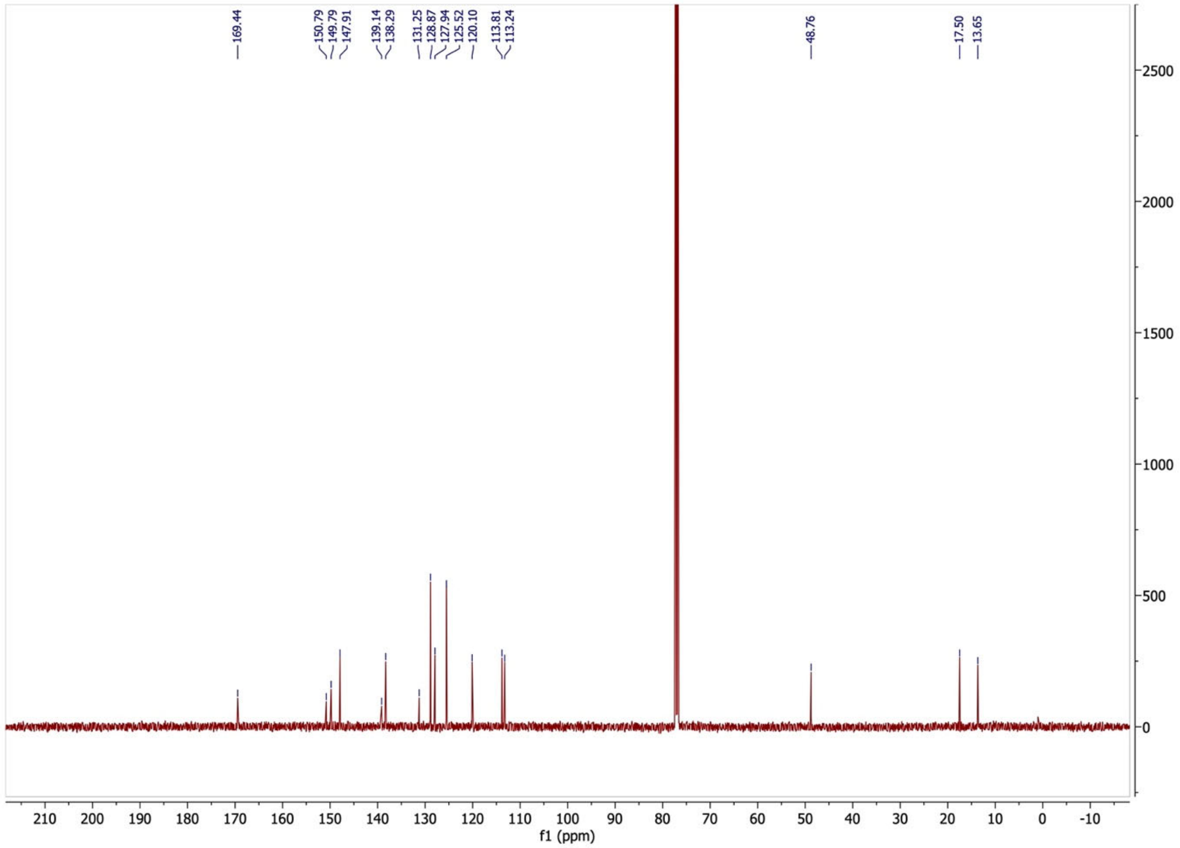

S34: ^{13}C NMR spectrum of (*R*)-2-((3-methyl-1-phenyl-1*H*-pyrazol-5-yl)thio)-*N*-(pyridin-2-yl)propenamide (**7q**).

(*S*)-2-((3-methyl-1-phenyl-1*H*-pyrazol-5-yl)thio)-*N*-(pyridin-2-yl)propenamide (7r)

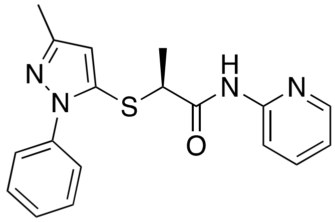

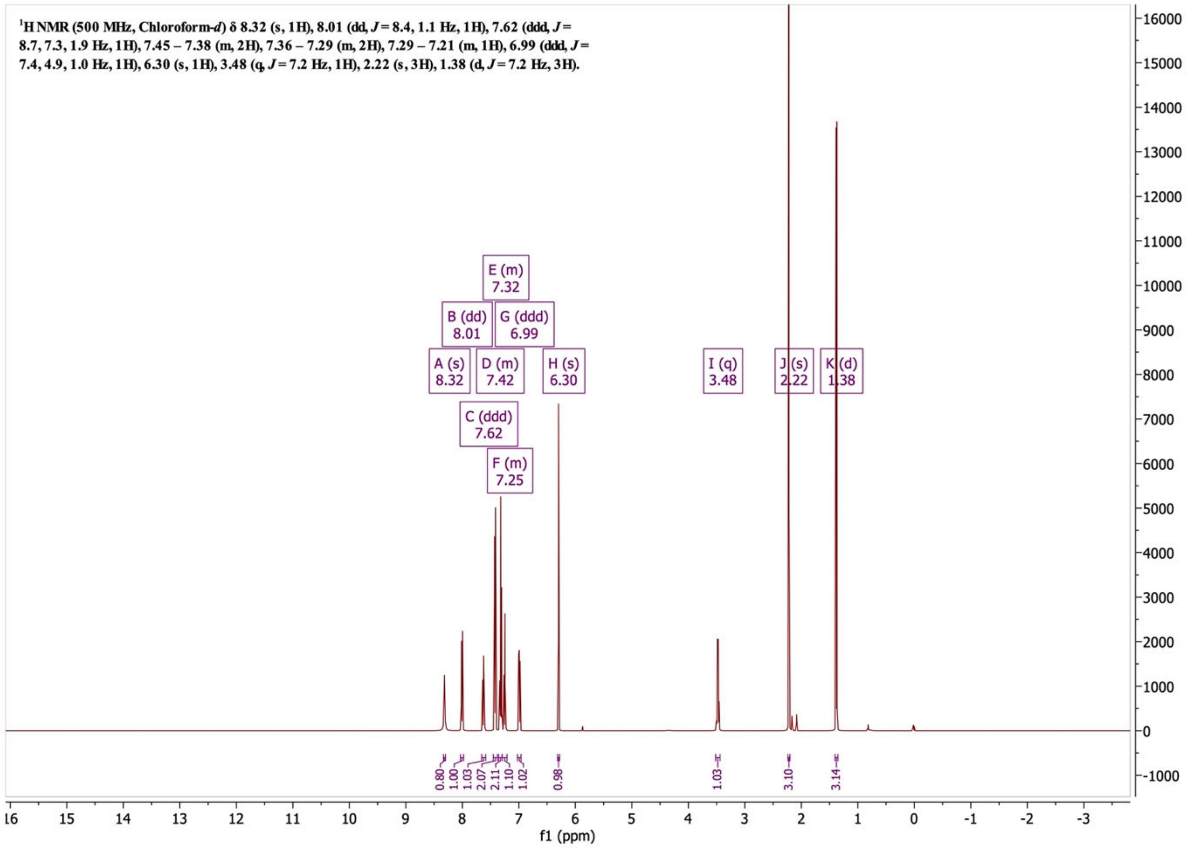

S35: ^{1}H NMR spectrum of (*S*)-2-((3-methyl-1-phenyl-1*H*-pyrazol-5-yl)thio)-*N*-(pyridin-2-yl)propenamide (**7r**).

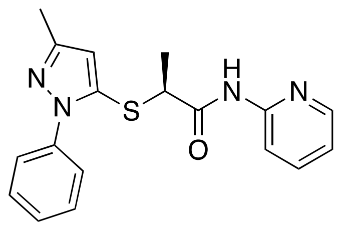

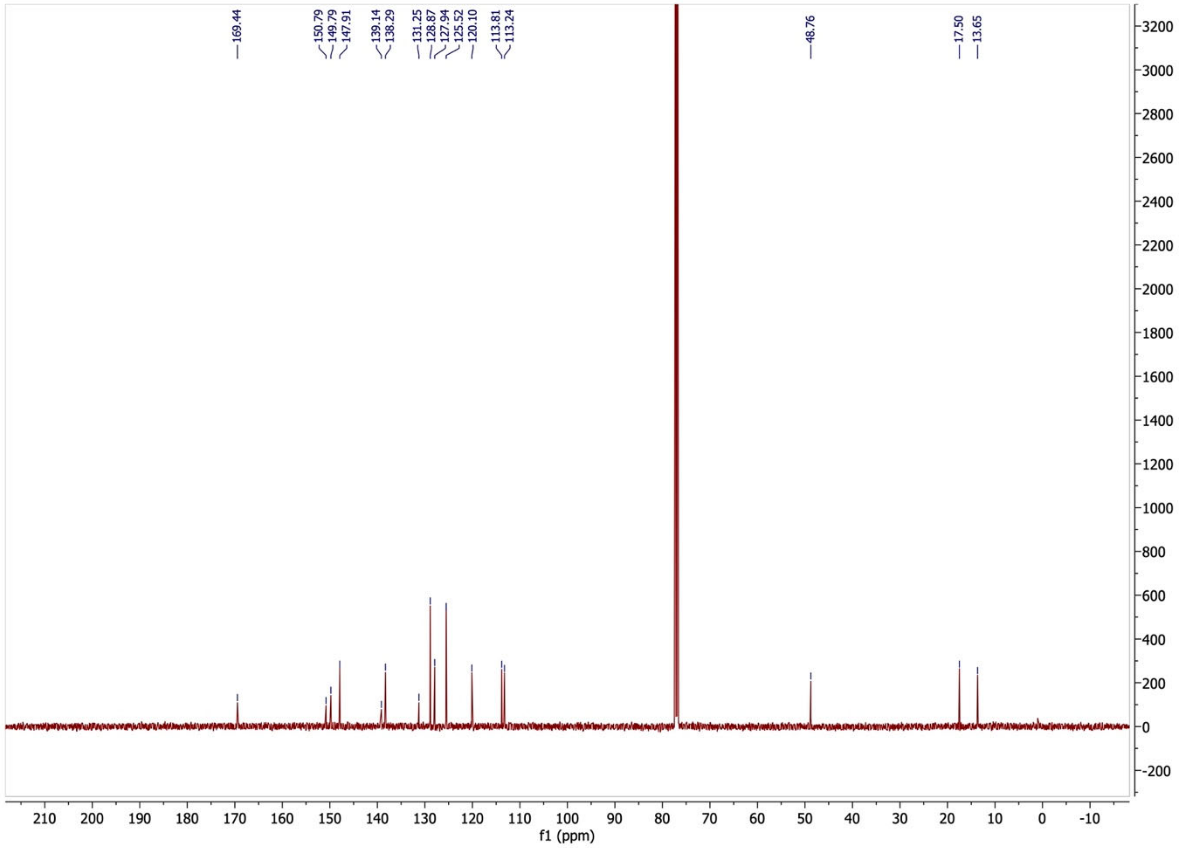

S36: ^{13}C NMR spectrum of (*S*)-2-((3-methyl-1-phenyl-1*H*-pyrazol-5-yl)thio)-*N*-(pyridin-2-yl)propenamide (**7r**).

Enzymatic Assays

Inhibition Graphs:

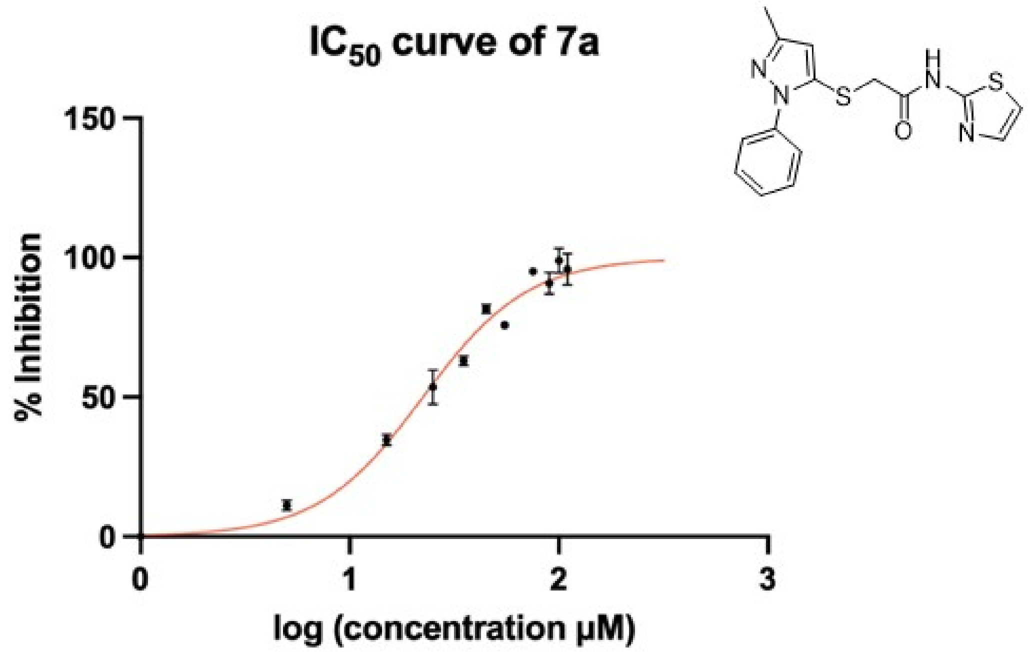

IC_{50} = 22.4 +/- 4.9 μM

S37: Inhibitory graph of pyrazole **7a**.

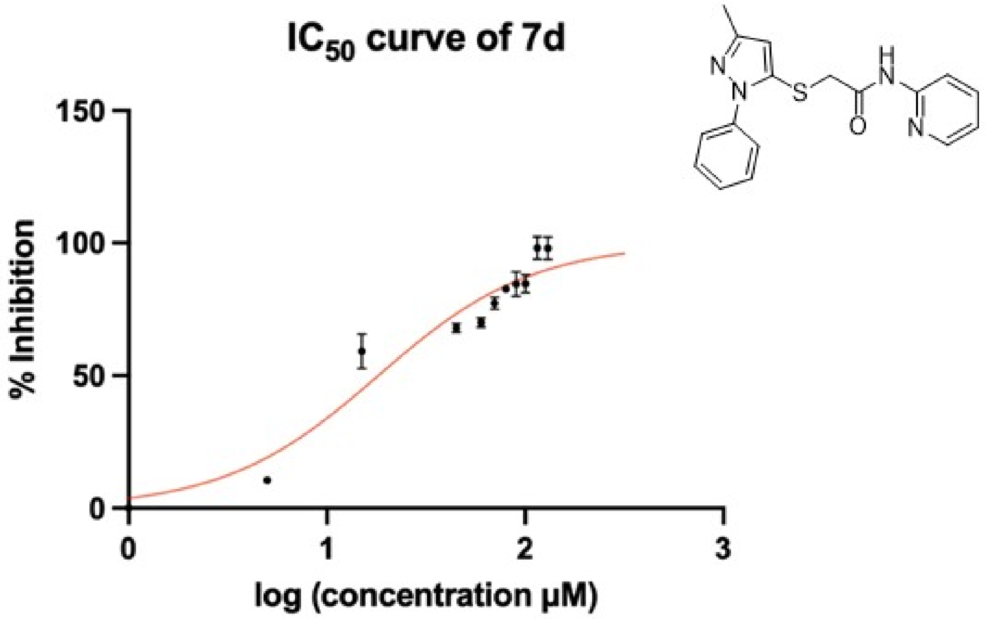

IC_{50} = 17.9 +/- 8.0 μM

S38: Inhibitory graph of pyrazole **7d**.

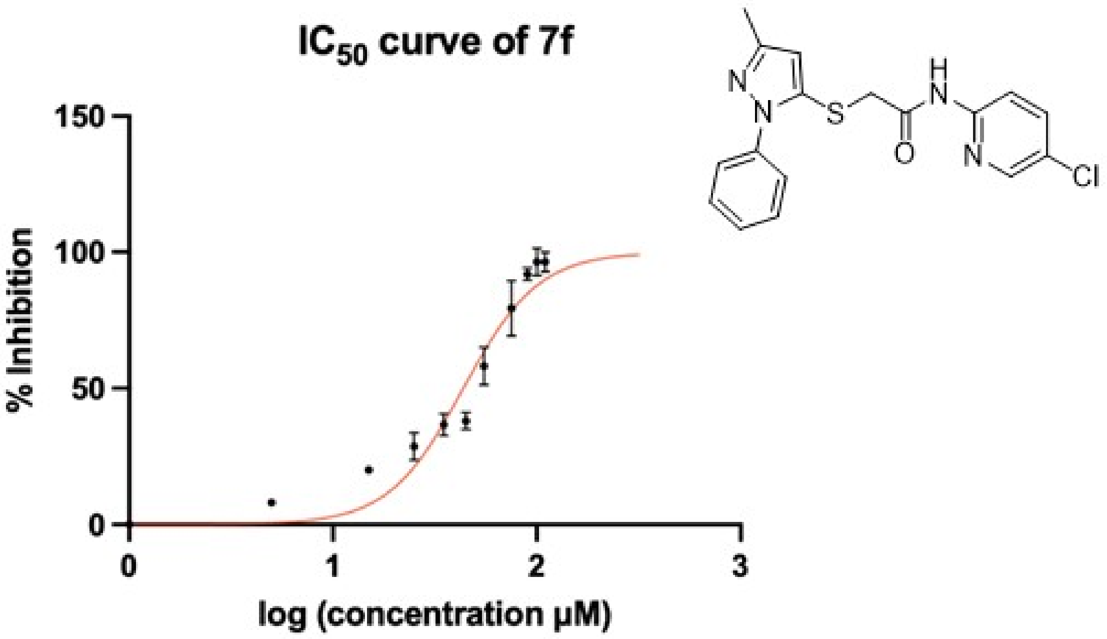

$IC_{50} = 43.6$ +/- 9.1 μM

S39: Inhibitory graph of pyrazole **7f**.

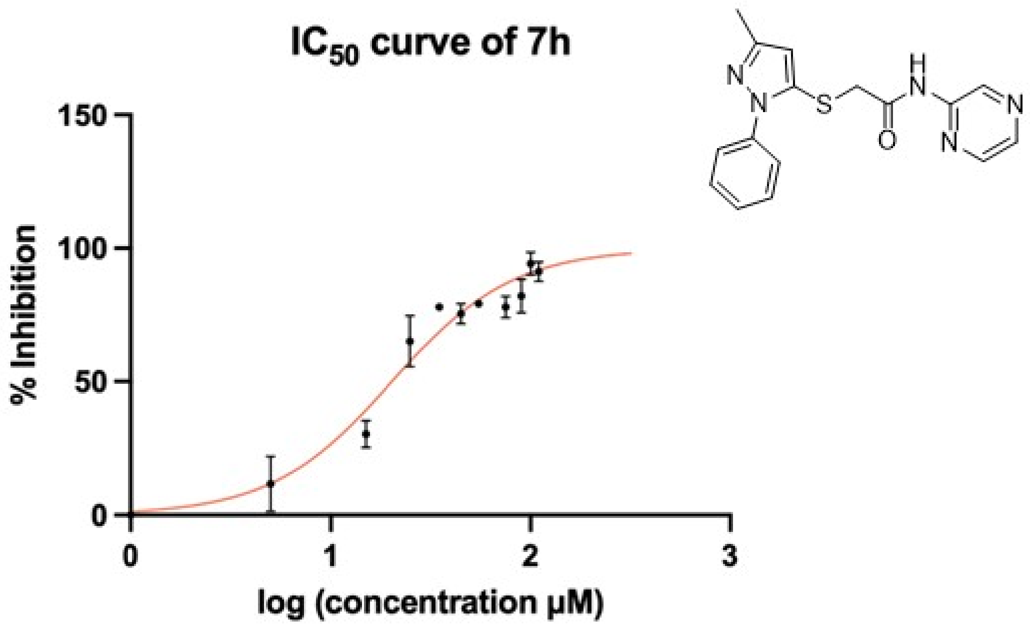

$IC_{50} = 20.2$ +/- 6.8 μM

S40: Inhibitory graph of pyrazole **7h**.

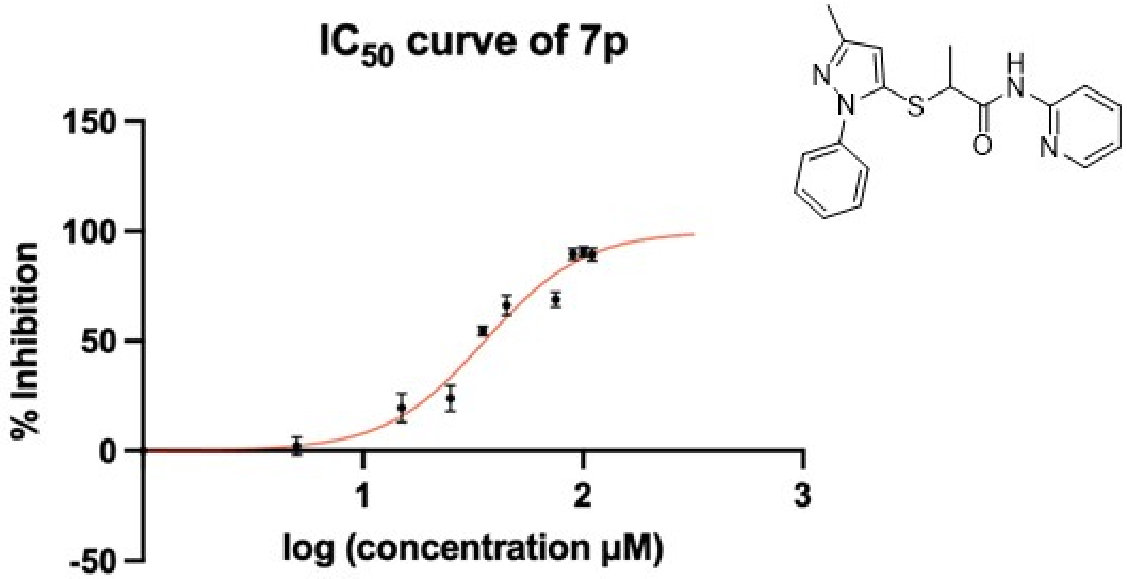

$IC_{50} = 35.7$ +/- 6.8 μM

S41: Inhibitory graph of pyrazole **7p**.

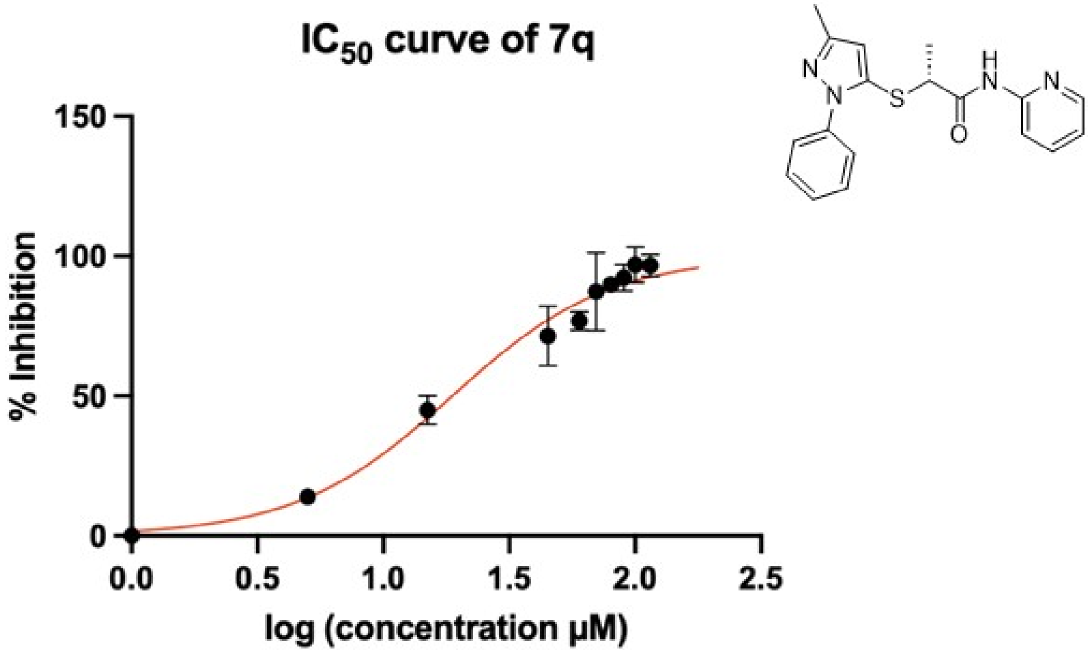

$IC_{50} = 18.8$ +/- 4.3 μM

S42: Inhibitory graph of pyrazole **7q**.

Thermal Shift Assay K_i Graphs:

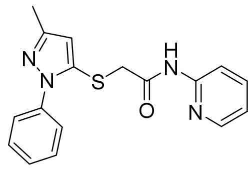

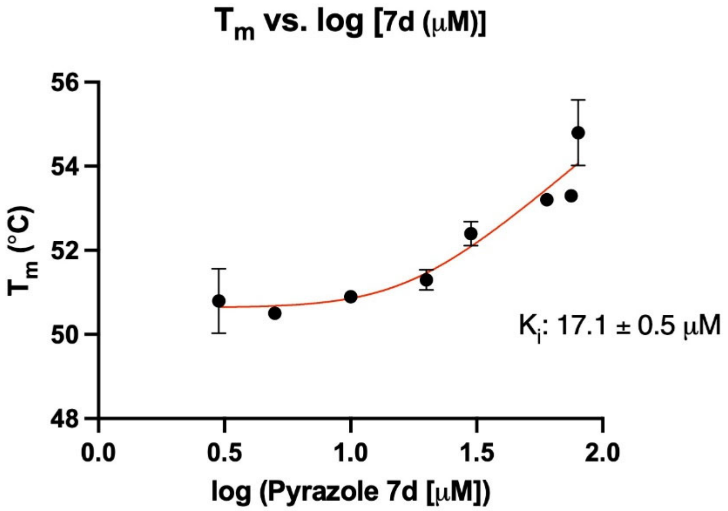

S43: Thermal shift plot of Pyrazole **7d**.

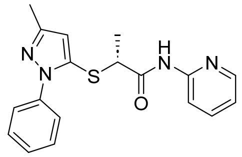

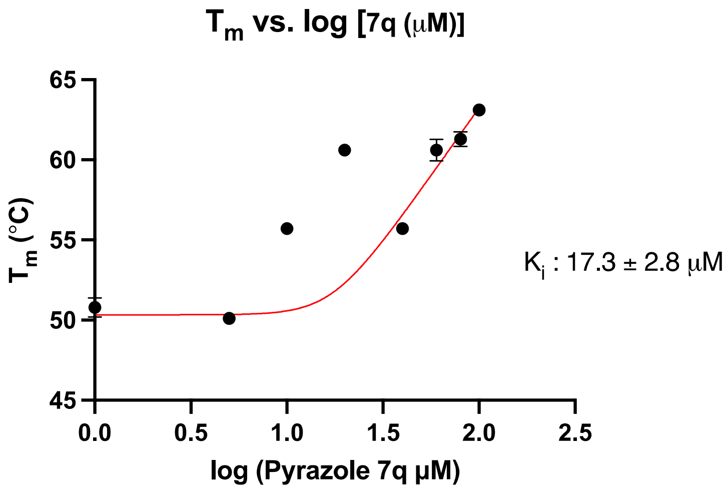

S44: Thermal shift plot of Pyrazole **7q**.
